# Global Characterization of Commercial Generation 0–7 Poly(amidoamine) Dendrimers: Challenges and Opportunities for Analysis

**DOI:** 10.1021/acsomega.5c07827

**Published:** 2025-10-01

**Authors:** Owen Tooley, William Pointer, Rowan Radmall, Steve Huband, James Town, Anne Martel, Patricia Monteiro, Thomas Floyd, Paul Wilson, Daniel Lester

**Affiliations:** † Department of Chemistry, 2707 University of Warwick, Coventry CV4 7AL, U.K.; ‡ X-Ray Diffraction RTP, 2707 University of Warwick, Coventry CV4 7AL, U.K.; § Polymer Characterization RTP, 2707 University of Warwick, Coventry CV4 7AL, U.K.; ∥ 56053 Institut Laue-Langevin (ILL), 71 Av. des Martyrs, Grenoble 38000, France; ⊥ Advanced Drug Delivery, Pharmaceutical Sciences, 468087 R&D, AstraZeneca, Cambridge CB2 0AA, U.K.; # Advanced Drug Delivery, Pharmaceutical Sciences, 468087 R&D, AstraZeneca, Macclesfield SK10 2NA, U.K.

## Abstract

PAMAMs make up the most ubiquitous family of dendrimers in use today, with their NH_2_ surface functionality rendering them an excellent choice for several valuable applications. However, impurities introduced during synthesis and their surface functionality present unique challenges for analysis. In this work, we discuss and review the applicability of a range of analytical techniques and present methodologies to analyze PAMAM dendrimers from generation 0–7, to extract both chemical and size-based information, through these data, we clearly show how molecular weight and size grow through the generations. Additionally, we show how the shape factor changes from close to 1 in low generations, indicating a majority of mass in the shell of the system, to values around 0.75, indicating uniformly distributed mass across the system. We further highlight where challenges in analysis can occur, and strategies that can be employed to yield optimum results. These results are then explored in the context of the analytical systems employed, in a first-of-its-kind study to provide guidance on the best systems to choose for a variety of analytical applications.

## Graphical abstract

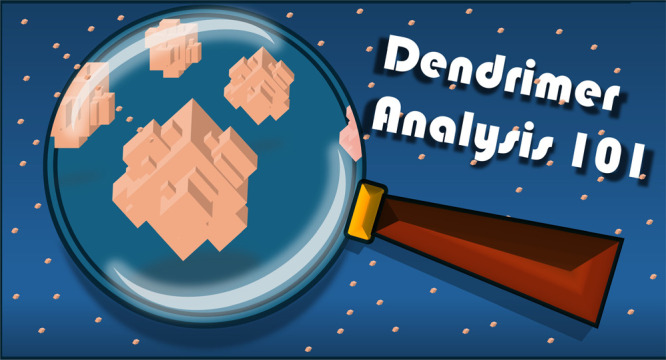

## Introduction

Since their initial synthesis in 1978,^1^ dendrimers, highly branched and ordered polymeric particles, have been of great interest for a variety of applications, including the binding of ionic guest molecules, inclusion of small molecules, and host–guest interactions. While the first dendrimers were based on ethylene diamine repeating units, subsequent work introduced alternative dendrimer families, mainly those based on lysine and *N*-(2-aminoethyl) acrylamide repeating units. The latter of which have come to be known as PAMAM, or Starburst dendrimers.^2–4^

Dendrimers are synthesized through repetitions of identical reactions, ideally resulting in monodisperse macromolecules. Each repetition of the reaction represents a “generation” of the dendrimer. There are two synthetic strategies employed to accomplish this: either convergent or divergent syntheses. While literature discussing the merits and drawbacks of these different synthetic strategies is plentiful, broadly, convergent polymers are more costly to produce, but of higher purity, whereas divergent polymers are cheaper to produce but come with lower purity.^5,6^

The most widely studied family of dendrimers is poly­(amido amine) (PAMAM) dendrimers, first synthesized by Tomalia et al.^4^ Typically produced through divergent synthesis, characterization of these dendrimers proves challenging due to structural complications with the potential for multiple structurally similar impurities existing simultaneously within the overall dendrimer.

The majority of commercially available PAMAM dendrimers are synthesized from an ethylenediamine (EDA) core, followed by successive additions of methyl acrylate and EDA, yielding both half- and full generations, respectively. However, there exists the potential for excess unremoved EDA to produce brand-new dendrimers during the methyl acrylate addition, resulting in a lack of control over dispersity during synthesis. Intramolecular addition occurs via an arm-to-arm cyclization and is problematic, as it prevents the exponential growth of the dendrimer through subsequent generations, limiting the amount of available primary surface amines. This type of impurity can become particularly prevalent in higher generational dendrimers due to more arms available to intramolecularly react. Intermolecular addition occurs when two dendrons cross-link, causing a dramatic increase in the molecular weight of the resulting product. If these impurities arise at earlier stages of the synthesis, the impurities can readily proliferate throughout subsequent additions; this may present as a high molecular weight tail in analysis through sizing techniques.

Characterization of PAMAM dendrimers is of paramount importance for several of their potential applications. Molecular weight, structure, and size must be known and carefully controlled for use in pharmaceutical applications, while both purity and chemical information are required for use as oil and fuel additives. Generally, characterization techniques can fall into one of two categories: chemical information or size information. Several different techniques for both areas have been used in the study of PAMAM dendrimers; however, studies have generally been limited in scope, and comparisons between techniques have not been fully explored. Herein, we provide a global characterization approach to PAMAM dendrimer generations, G0–7, exploring both techniques that have been successful and those that struggle to provide information on use.

## Experimental Section

### Materials and Methods

G0–7 PAMAM dendrimers, acetic acid, citric acid, sodium citrate dihydrate, methanol, methanol-d4, deuterium oxide, trifluoroacetic acid (TFA), acetonitrile, sodium chloride, bovine serum albumin (BSA), lysozyme, 2,5-dihydroxybenzoic acid, and α-cyano-4-hydroxycinnamic acid were purchased from Merck. Phosphate buffer tablets were purchased from Fisher Scientific. Poly­(ethylene glycol) was purchased from PSS. The G0–7 PAMAM dendrimers were supplied as solutions in methanol, dialyzed against ultrapure water, and subsequently freeze-dried prior to use. All of the chemicals were used as supplied.

### Nuclear Magnetic Resonance Spectroscopy

All samples were dissolved at a concentration of 25 mg mL^–1^ in methanol-d4 before being transferred to a standard 5 mm NMR tube. Nuclear magnetic resonance (NMR) spectra were acquired on either a Bruker Avance III 400 MHz or Bruker Avance HD 400 MHz spectrometer. Data was processed in Bruker Topspin 4.1.4 software.

### Diffusion-Ordered Spectroscopy NMR

All samples were dissolved at a concentration of 10 mg mL^–1^ in deuterium oxide (D_2_O) before being transferred to a standard 5 mm NMR tube. Diffusion-ordered spectroscopy (DOSY) NMR spectra were acquired on a Magritek Spinsolve 80 Carbon Benchtop NMR spectrometer equipped with a *z*-axis gradient coil capable of generating a maximum field gradient of 500 mT/m. DOSY spectra were interpreted using Magritek Spinsolve version 2.3.6 or the GNAT, version 1.3.2, developed by the Manchester NMR Methodology Group.^7^

### Infrared Spectroscopy

Infrared (IR) measurements were performed on an Agilent Cary 630 IR with an ATR sample cell. Samples were run from 650 to 4000 wavenumbers for 16 scans in absorbance mode. Prior to measurement, samples were dissolved in a minimum amount of methanol and the resultant solution pipetted onto the ATR crystal.

### Ultraviolet–Visible Spectroscopy

Ultraviolet–visible (UV–vis) spectra were recorded on an Agilent Cary 60 UV–vis using a quartz cuvette with 10 mm optical length within the range 200–800 nm, calibrated using methanol as a blank solution. The PAMAM Dendrimer solutions were diluted first to a concentration of 1 mg mL^–1^ in methanol and then further diluted to a final concentration of 0.1 mmol in methanol for measurement.

### Dynamic Light Scattering and Zeta-Potential

Dynamic light scattering (DLS) measurements were carried out on an Anton Paar Litesizer 500 in methanol, with a 1 mm path length cuvette, for 60 runs. A refractive index (RI) of 1.45 and number weighting were used during particle size calculation.

ζ-potential measurements were carried out on an Anton Paar Litesizer 500, using an Anton Paar omega cuvette over a maximum of 1000 runs. The measurements were carried out in 0.1 M, pH 3.0, citrate buffer. Samples were measured at a concentration of 5 mg mL^–1^.

### High-Performance Liquid Chromatography

High-performance liquid chromatography (HPLC) was performed by using an Agilent 1260 HPLC system equipped with a photodiode array (PDA) detector. The method was based on the work by Islam et al.^8^ The detection wavelength was 210 nm. Mobile phase A was water with 0.14% TFA, and mobile phase B was acetonitrile with 0.14% TFA. The injection volume was 80 μL. Separation was achieved using a Discovery BIO Wide Pore C5 (10 μm) HPLC Column (250 × 4.6 mm) held at 30 °C. Gradient elution was used to separate the samples, with the gradient as 5% organic for the first 10 min, followed by a linear increase to 95% organic over 29 min, and finally returning to 5% organic over 1 min. Samples were dissolved at a concentration of 5 mg mL^–1^ in 100% mobile phase A.

### Gel Permeation Chromatography

Gel permeation chromatography (GPC) data were obtained using a Malvern Panalytical OMNISEC system using a PSS NOVEMA (300 × 7.8 mm, 10 μm, 1000 Å) and a PSS NOVEMA (300 × 7.8 mm, 10 μm, 100 Å) in series. The eluent used was 50 mM sodium chloride and 0.1% (v/v) acetic acid. The system was calibrated with a single Malvern Panalytical PEO 24 kDa narrow standard, and absolute molar masses were determined using the OMNISEC software V11. Samples were run at a concentration of 1 mg mL^–1^ in ultrapure water. OMNISEC software, version 11.32, was used.

### Asymmetric Field Flow Field Fractionation

Asymmetric field flow field fractionation (AF4) measurements were performed on a Postnova AF2000 MultiFlow FFF system, running NovaFFF AF2000 version 2.2.0.1 and equipped with an RI and multiangle light scattering (MALS) detector. Separation was performed using an analytical FFF channel (300 × 60 × 40 mm).^9^ The injection volume was 20 μL, and the eluent was 0.9% NaCl in ultrapure water. The detector flow was 0.5 mL min^–1^. During each run, sample injection took place over 4 min, after which there was an initial 3 min focusing step with a 3 mL min^–1^ cross-flow. The elution step then took place over 40 min, with a constant 3 mL min^–1^ cross-flow for the first 30 min, followed by a linear cross-flow decrease from 3 to 0 mL min^–1^ over the next 5 min, and then finally 0 mL min^–1^ cross-flow for the final 5 min. After each run, a rinse step of 0.2 mL min^–1^ for 0.5 min was used. Samples and calibrants were dissolved at a concentration of 5 mg mL^–1^ in the eluent. AF4 fractograms were analyzed using NovaFFF AF2000 2.2.0.1. The system was calibrated against the monomer and dimer of BSA. Particle sizing used a Zimm calculation type,^10^ and d*n*/d*c* = 0.240.

### Matrix-Assisted Laser Desorption Ionization Time of Flight Mass Spectrometry

Matrix-assisted laser desorption ionization time of flight mass spectrometry (MALDI-TOF-MS) spectra were acquired on a Bruker Ultraflex Extreme MALDI TOF/TOF spectrometer in linear or reflector mode, equipped with a nitrogen laser delivering 2 ns laser pulses at 337 nm, with positive ion TOF detection performed using an accelerating voltage of 25 kV. The matrix solution was a 1:1 mixture of 2,5-dihydroxybenzoic acid and α-cyano-4-hydroxycinnamic acid dissolved in a 3:1 mixture of acetonitrile: water at 15 mg mL^–1^. Samples and calibrants were dissolved in a 3:1 mixture of acetonitrile: water at 1 mg mL^–1^. Sample spots on the MALDI plate were produced by spotting 0.5 μL of matrix solution, allowing this to dry, and then spotting 0.5 μL of sample onto the dried matrix spot. This was then dried, and a final 0.5 μL of matrix solution was added onto the dried spot. PAMAM G1 and G2 were calibrated against poly­(ethylene glycol) 2000, and PAMAM G3 was calibrated against poly­(ethylene glycol) 8000. PAMAM G4 and G5 were calibrated against the 1+ and 2+ peaks of lysozyme, PAMAM G6 was calibrated against the dimer peak of lysozyme, and PAMAM G7 was calibrated against the monomer and dimer peaks of BSA.

### Small Angle X-ray Scattering

Small-angle X-ray scattering (SAXS) measurements were made using a Xenocs Xeuss 2.0 equipped with a microfocus Cu Kα source collimated with scatterless slits. The scattering was measured using a Pilatus 300k detector with a pixel size of 0.172 × 0.172 mm. The distance between the detector and the sample was calibrated using silver behenate (AgC_22_H_43_O_2_), giving a value of 0.547 m. The q range for the detector was 0.02 and 0.7 Å^–1^. An azimuthal integration of the 2D scattering profile was performed using Xenocs Xsact software version 2.4, and the resulting data were corrected for the absorption, sample thickness, and background.^11^ Samples were dissolved to a concentration of 5 mg mL^–1^, mounted in 1 mm thick borosilicate glass capillaries, and each sample was measured for 4 h. The Irena SAXS analysis package was used for modeling the SAXS data.^12^ The particles were modeled as spheres with a radius given by a Gaussian distribution. The distribution was modeled as a volume distribution.

### Small-Angle Neutron Scattering

Small-angle neutron scattering (SANS) measurements were made at the D22 beamline at the Institut Laue-Langevin (ILL). All samples were made up to a concentration of 5 mg mL^–1^ in methanol-d4. Before being transferred to 2 mm Suprasil quartz Hellman cells for measurement at 25 °C. The first detector was at 8 m, and the second detector at 1.4 m. The neutron wavelength was 6 ± 10% Å. Measurements were taken for 60 min. The raw data were radially averaged, corrected for electronic background, empty cell and methanol-d4 scattering, and normalized by transmission, thickness, and incident beam flux, using GRASP (ref: 10.1107/S1600576723007379). Fitting was performed in SASView 5.0.6 using the DREAM algorithm with 100000 steps.

## Results and Discussion

### NMR Spectroscopy

The workhorse of organic molecule characterization is undoubtedly NMR spectroscopy. With a variety of nuclei and pulse sequences available, the information available from NMR is plentiful and has been previously used widely for studying PAMAM dendrimers.^4,13^

Initially, G0 PAMAM is representative of that of a highly symmetric small molecule with distinct splitting and discernible coupling constants (Figure 1). These environments are essentially identical as the generation increases; however, due to subtle differences in each subsequent generation, the evolving peaks become broad and lose resolution, to the point that in G7, only 5 broad peaks from the dendrimer can be observed. The assignments were made using a combination of relative integrals, chemical shifts, and 2D experiments (Figures S1 and S2).

**Figure 1:**
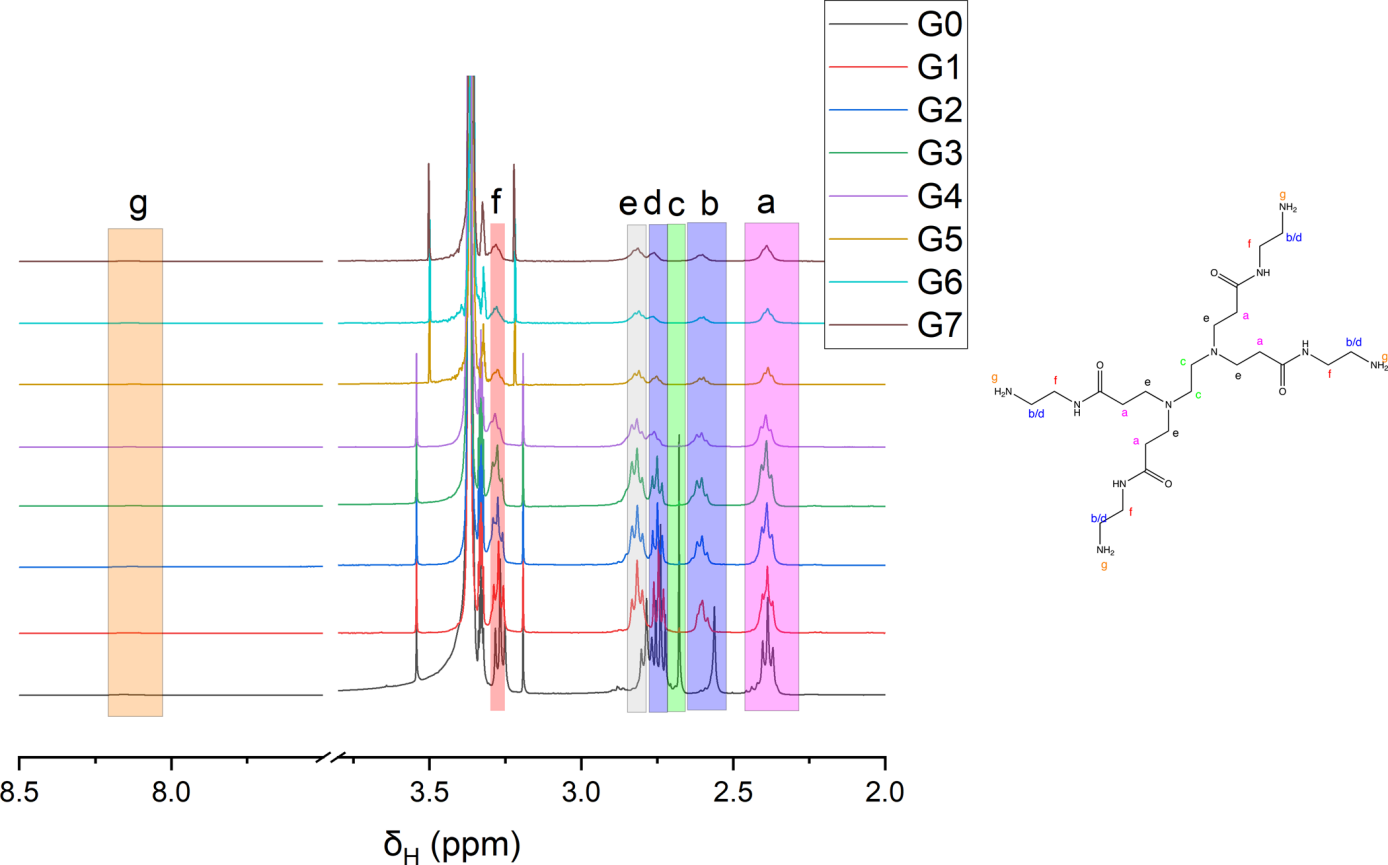
^1^H NMR spectra of PAMAM G0–7.

The relative peak area for the proton environment at 2.69 ppm (H^c^) can be assigned to the methylene environments in the ethylene diamine core. The relative intensity of this signal significantly decreases as the dendrimers grow, which is to be expected as this environment corresponds to the EDA core, which is indicative of the diminishing fraction of H^c^ compared with the total number of protons in the divergent corona as the dendrimer grows. Additionally, two peaks can be observed for protons b/d. This is because one of the methylene environments is adjacent to the tertiary amine (H^b^) while the other is adjacent to the terminal amine (H^d^).^13^ The environment adjacent terminal amine (H^d^) can be particularly useful in reaction monitoring scenarios as it will show substantial chemical shift changes upon functionalization of the primary amine and thus can be used to monitor the degree of modification.

Carbon-13 NMR provides somewhat less useful data, as all spectra appear alike (Figure S3). Two peaks of note in the ^13^C spectra, however, are the peak at ∼174 ppm. This corresponds to the quaternary carbon of the amide group, an environment that is obviously absent from the ^1^H spectra. Likewise, the peak at ∼51 ppm corresponds to the EDA core of the dendrimer, hence its apparent decrease in size relative to the other peaks upon generational growth. Further structural elucidation was performed by using ^1^H–^13^C HSQC experiments (Figure S2).

#### Diffusion Ordered Spectroscopy

An additional NMR experiment of use for polymeric nanoparticles is diffusion-ordered spectroscopy (DOSY), which allows the measurement of a molecule’s diffusion constant, and the diffusion constant can then be used to calculate the hydrodynamic radius of the particle through the Stokes–Einstein Equation (vide infra, Table 2).

The change in diffusion constant correlates with the increase in molecular size, with a 10-fold decrease in diffusion constant from G0 (6.92 × 10^–10^ m^2^ s^–1^) and G7 (0.71 × 10^–10^ m^2^ s^–1^). This clear change in diffusion constant provides a useful tool for monitoring uptake of functional ligands, while the ligand uptake itself will decrease the diffusion constant of the PAMAM. Due to the increased mass, the ligand itself will also have a very large decrease in diffusion constant, which can be used for kinetic profiling of a reaction.^14,15^

Recent work published by our group also demonstrates that through a universal calibration, molecular weights can be calculated based on diffusion constants and solvent viscosity.^16^ This technique directly measures the hydrodynamic radius of the analyte and then uses a solvent-independent universal calibration to calculate the molecular weight.^14,16,17^ Once again, it can be seen in these results that there is a clear trend in the molecular weights, with increasing molecular weight as the generation increases. (Figure S5) However, it is noteworthy in this data that while there is excellent agreement for dendrimers G0–3, the technique hugely underestimates the molecular weight for higher generations. The reason for this is likely due to the universal calibration used in the MaDDOSY technique. As can be seen in the original work, most polymers tested in the calibration are linear GPC standards, which will take on very different geometries in solution than dendrimers, which are likely to be significantly denser due to their high degree of branching, resulting in significantly lower hydrodynamic radii than an equivalent linear polymer of the same molecular weight.

#### IR Spectroscopy

IR spectra are available in the Supporting Information, including those conducted in methanol and featuring the methanol background subtracted (Figures S6 and S7). Signals at approximately 1640 and 1550 cm^–1^ correspond to the stretching mode carbonyl of the tertiary amide and the bending mode of the primary amine, respectively. Notably absent from the spectra are the expected amine stretches associated with the primary amine; this is likely due to this vibration overlapping with the broad alcohol stretching of the methanol, observed at 3610–3040 cm^–1^ and therefore being subtracted with the buffer. A key use of PAMAM dendrimers results from the ability to conjugate other molecules of interest to the primary amine surface, as such IR spectroscopy is a valuable tool to monitor this reaction—there should be a clear decrease in the primary amine vibrations because of conjugation, and so this would form a useful reaction monitoring tool for any future work.

#### UV–Vis Spectroscopy

Limited UV–vis Spectroscopy of PAMAM dendrimers has been previously reported^18,19^ ; however, full spectra of PAMAM G0–7 in methanol at 0.1 mM concentration are available in the Supporting Information (Figure S8). There are two peaks of interest: a large peak at 200–260 nm, where the maximum absorbance displays a bathochromic shift as the dendrimer increases in size, and a smaller peak between 260 and 320 nm, with a consistent λ_max_ at 280 nm. The primary peak has a clear extinction coefficient increase as the dendrimer size increases, from approximately 15,000 M^–1^ cm^–1^ for G0 to over 25,000 M^–1^ cm^–1^ for G7. This peak arises from a π–π* transition from the amide carbonyl. Peak broadening as the dendrimer grows can be observed here, likely due to a wider range of energy levels being present, arising from an increased number of carbonyls present. The secondary peak at 280 nm also shows a hyperchromic shift as generation increases, and this peak is the result of the n−π* transition of the amines in the structure. This 280 nm peak also provides a useful opportunity for reaction monitoring as the amines partially responsible for this absorbance are the primary amines at the surface of the dendrimer, which will change upon reaction.

#### DLS and Zeta-Potential

DLS studies of both native and functionalized PAMAM dendrimers are well-known^20–22^ ; however, these studies typically employ dendrimers of G4 or greater. While DLS can be used for particles as small as 0.1 nm in diameter, in practice, samples with particles smaller than approximately 5 nm prove difficult to analyze due to their incredibly poor scattering and the tendency for large particles, even at low concentrations, to dominate the scattering profile.^23^ For G4-G7, DLS demonstrates its utility, with sizes shown in Table 2 and distributions available in the Supporting Information (Figures S9–S11), being capable of easily distinguishing between the different sizes of dendrimer. Of course, the pH and ionic strength of the system can change the degree of protonation of the amines within the dendrimer structure and, therefore, change the size. This is well understood, and therefore, the DLS measurements have been conducted in pure methanol. This is because the p*K*_a_ of the PAMAM dendrimer primary amines is at a value of 6.85,^24^ compared to the methanol at 15.3, protonation of the PAMAM is extremely unlikely, and electronically unfavorable, and therefore the PAMAM is likely to remain in a net neutral charge in pure methanol. Of course, being at a net neutral charge does mean there is potential for the dendrimers to aggregate in solution,^25^ which can cause reproducibility issues within DLS. This can be alleviated by using high ionic strength systems; however, this would prevent comparison between other techniques in this work. Additionally, reactions involving PAMAM dendrimers are rarely performed in high ionic strength solvents, and therefore, the utility of such measurements in this work is limited. Furthermore, for G0–3, we have seen very poor reproducibility between DLS measurements; this can be explained when looking at the correlation functions for these samples (Figure S9). With noisy decay plots and relatively poor G_2_ decay, it becomes very difficult to fit the correlation function and therefore determine a hydrodynamic radius. While traditionally the way to improve this would be to run at a higher concentration, higher concentrations are likely to cause even more aggregation effects, and therefore are unlikely to improve the resultant data. Therefore, the utility of DLS for PAMAM systems should be limited to G4 and greater.

Zeta-potential measurements are an important and straightforward measurement to characterize the solution stability of a nanoparticle, a property that is crucial to several applications of dendrimers.^26^ The zeta potential measurements for PAMAM G0–7 are shown in Table 2, and full plots are available in the Supporting Information (Figure S12). Due to the primary amine surface of PAMAM dendrimers, they are incredibly susceptible to changes in surface charge based on pH and thus protonation of the surface amines. To keep the surface amines in one protonation state, citrate pH 3.0 buffer (0.1 M) was used, and as such, the ζ-potential measurements for each dendrimer are weakly positive. The values of the ζ potential are broadly similar across the generations, except for G0, which is nearly neutral. However, this is likely due to the small size and limited particulate nature of the G0 dendrimers. The values also indicate poor to moderate stability in solution, which suggests that the particles may tend to aggregate, a phenomenon which has been greatly studied in existing literature;^25,27,28^ however, the stability can be greatly improved with surface modification, and the resultant ζ-potential can be greatly affected.^29^

### High-Performance Liquid Chromatography

The techniques discussed thus far prove useful for monitoring the chemical environment of the dendrimers; however, they all struggle to characterize the impurities previously described due to impurities possessing similar chemical environments to the pure dendrimers. HPLC can be useful in these cases, as often impurities such as cyclization or uneven chain growth will cause differences in the surface chemistry, which will, in turn, cause differences in column interaction and retention times. This has been shown in the work by Islam et al., where different surface chemistries produced markedly different retention times.^8^ This work also produced a reliable HPLC method for the separation of different generations of PAMAM (Figure 2)

**Figure 2:**
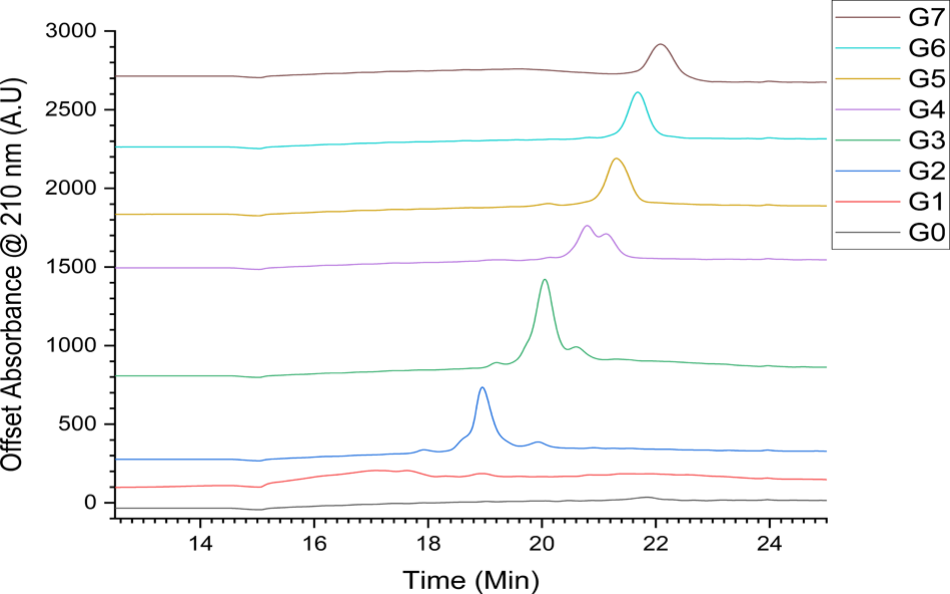
HPLC chromatograms of PAMAM G0–7 at 210 nm.

The traces for G0 and G1 show no discernible peaks, likely because there was no retention of the molecules, and elution occurred with the solvent front. However, the increase in retention time as the dendrimer generation increases from G2 onward shows that this can be an effective method for separating dendrimers of different generations. G2–5 possess smaller impurity peaks flanking the parent dendrimer peak. In G3 and G4, peaks with higher retention times are present, which overlay with the main peaks in G4 and G5, respectively; these impurities likely come from intermolecular coupling during the synthesis. Meanwhile, in G3, G4, and G5, there are peaks present at lower retention times, which may instead be caused by trailing generations being present from the unreacted ethylene diamine between samples. In G6 and G7, these impurities are not present, which is likely due to the large differences in size between the target dendrimer and impurities, allowing for easier separation during the synthesis.^4,30,31^ However, this may also be due to the impurities showing retention times similar to those of the parent dendrimer, as can be seen by the coalescence of the two peaks in generation 4. The dendrimer purity by mathematical peak area was also calculated (Table S3) and shows a general trend of increasing as dendrimer generation increases, from a purity of ∼85% for G2 up to a maximum of ∼95% for G7, as is to be expected due to the reasons previously discussed. The purities for G0 and G1 were not calculated because there were no discernible peaks in the chromatograms.

HPLC also provides a promising technique for the monitoring of changes to surface functionality during conjugation reactions, for the same reasons as previously discussed; these changes will affect the properties of the dendrimer surface and therefore the retention of the dendrimers on the column. This also provides opportunities for reaction monitoring, as the changes in retention from the native dendrimer, brought about by the decrease in surface amine groups, allow for calibration and therefore kinetic monitoring of the decrease in the dendrimer peak.

#### Gel Permeation Chromatography

The gold standard for macromolecular size characterization is GPC, providing information related to both molecular weight and molecular weight distribution. This can be incredibly useful for characterizing polydisperse systems, where there exists a significant variation in molecular weight, and sometimes architectures, which can be directly related to the macroscopic properties of the sample.^32^ Moreover, for narrow dispersity systems such as dendrimers, GPC can prove to be a useful tool for assessing their purity. The GPC chromatograms for PAMAM G0–7 are shown (Figure 3). Often, interactions with the column, particularly in aqueous conditions,^33^ can affect the retention of the analyte

**Figure 3:**
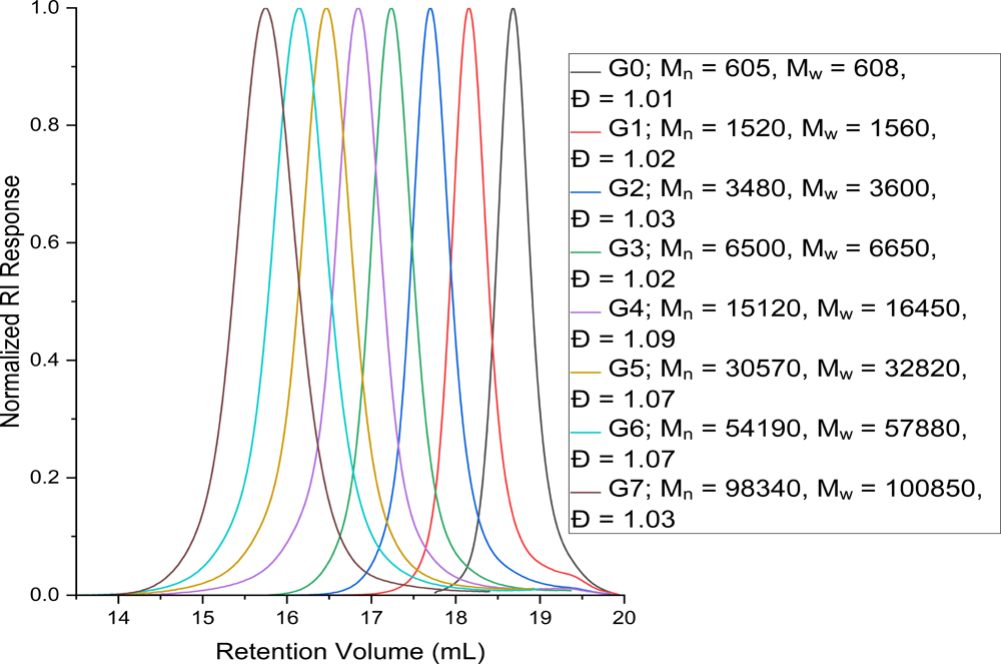
GPC Chromatograms for G0–7 PAMAM Dendrimers.

This is particularly prudent to mention with PAMAM dendrimers, which possess a positive charge under conditions such as those used in GPC, as shown through the zeta-potential measurements. To mitigate this, NOVAMA columns were used under acidic conditions, in which case both the columns and dendrimers were protonated, thus avoiding any column-analyte interactions.

Analysis of the dendrimers shows an expected increase in molecular weight with each subsequent generation, with the calculated molecular weight by MD-SEC closely matching the theoretical molecular weight up to G6. Furthermore, the determined dispersity for each generation is small, indicating largely monodisperse samples. The peaks here are symmetrical and have narrow dispersities, suggesting that there are few impurities present of vastly different sizes to the target molecule, and all species are broadly similar in molecular weight. A benefit of using GPC equipped with triple detection is the ability to calculate intrinsic viscosities and RI increments (d*n*/d*c*), values of which can be found in the Supporting Information (Table S5). MD-SEC provided a powerful tool for PAMAM characterization because traditional SEC calibration is often very dependent on the calibrant used, and often these calibrants are linear polymers that poorly replicate PAMAM solution behavior; in addition, by using MD-SEC, the true molecular weight is best calculated by light scattering, which MD-SEC allows. Further improvements may be made here by using conventional calibration using a range of narrow dispersity spherical standards, such as proteins, and comparing the results from conventional GPC and MD-SEC; however, this is outside of the scope of this work.

Figure 4 shows how the intrinsic viscosity changes through dendrimer generations—as explored and discussed by England et al.^34^ with other dendrimer types, the intrinsic viscosity increases from G0 to G4, before decreasing slightly and then plateauing for G5 to G7. This could be attributed to increasing dendrimer-solvent interaction as the dendrimer grows—owing to the increased presence of amine groups, before reaching an effective maximum at G4, when the hydration is such that the dendrimer size no longer affects the intrinsic viscosity. Intrinsic viscosity is, of course, dependent on the solvent conditions used and could be further explored. The d*n*/d*c* does not change with respect to the dendrimer size, with an arithmetic mean of 0.240.

**Figure 4:**
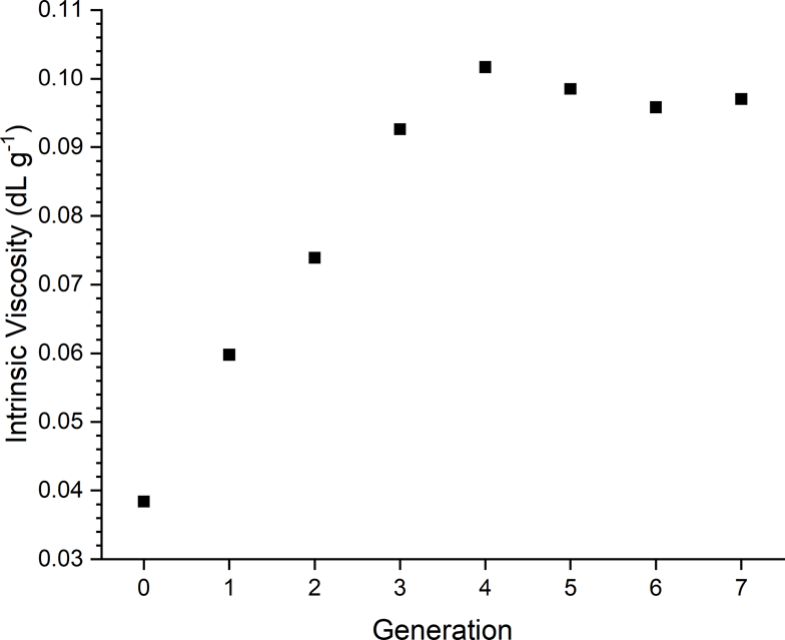
Intrinsic viscosity vs generation for PAMAM dendrimers in 50 mM sodium chloride +0.1% (v/v) acetic acid.

#### Asymmetric Field Flow Field Fractionation

AF4 is a further technique for determining the molecular weight of macromolecules and is becoming increasingly common in the study of therapeutic nanoparticles.^35^ Particle separation via AF4 occurs by way of a cross-flow, causing larger particles to elute more slowly than smaller particles. RI and multiple-angle light scattering (MALS) detectors were used to determine the molecular weights of the dendrimers. While MALS proves useful in terms of particle detection, it cannot be accurately used to determine the particle size of these dendrimers, as they are relatively small and therefore scatter isotropically, irrespective of angle. Previous work by Lee et al. has demonstrated AF4 to be a useful technique for the separation of higher-generation dendrimers^36^; however, with method development, we have been able to separate all dendrimers from G1 to G7. G0 could not be separated, as it is smaller than the minimum pore size membrane available (1000 Da) and, thus, is washed out of the fractionating channel.

Figure 5 illustrates the fractograms achieved for PAMAM dendrimers; as the separation is purely size-based, the difference between generations increases as the dendrimers get larger. Full fractograms are available in the Supporting Information (Figure S21). Additionally, similar to GPC, calibration can be used to calculate molecular weights (Table 1). As a purely size-based separation technique, the choice of calibrant here is of critical importance in the accuracy of the molecular weight calculations. Due to their compact spherical nature, PAMAM dendrimers, and indeed all dendrimers, share more similarities with globular proteins than they do with linear polymers in terms of their structure and, therefore, size in solution. For this reason, BSA is a good calibrant choice for this study and likely contributed to the good experimental values obtained. Similarly to MD-SEC and DLS, errors in the size information here are most prevalent in the smaller generations, where light scattering, as used in the MALS detection, is limited; however, as AF4 also allows size data to be obtained based on retention volume, these issues can be somewhat alleviated, making AF4 a good choice for the majority of PAMAM dendrimers.

**Figure 5:**
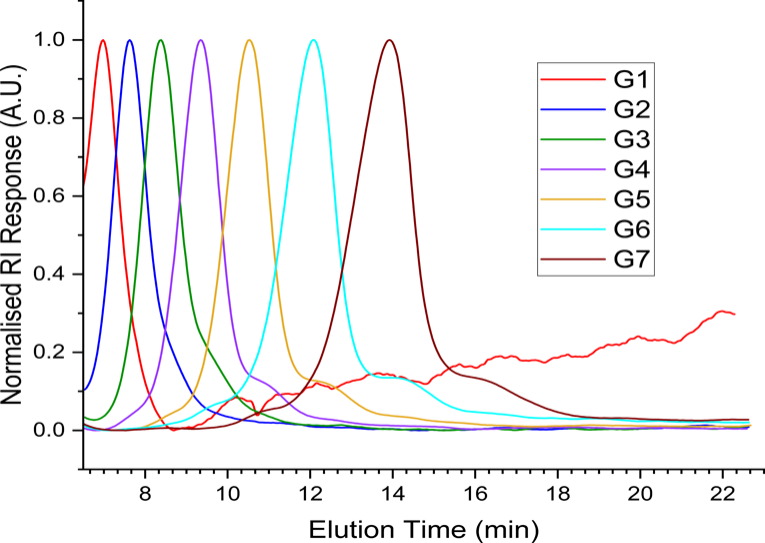
AF4 fractograms of PAMAM G0–7.

**Table 1:** Comparison of the PAMAM Molecular Weight Data across Multiple Techniques

sample	molecular formula	calculated *M*_wt_/g mol^–1^	MaDDOSY *M*_D_/g mol^–1^	GPC *M*_n_/g mol^–1^	AF4M_wt_/g mol^–1^	MALDI *M*_wt_/g mol^–1^
G0	C_22_H_48_N_10_O_4_	516.68	700	605		
G1	C_62_H_128_N_26_O_12_	1429.85	1500	1520	680	1453.0
G2	C_142_H_288_N_58_O_28_	3256.18	3000	3480	3940	3277.4
G3	C_302_H_608_N_122_O_60_	6908.84	4600	6500	6550	6982.6
G4	C_622_H_1248_N_250_O_124_	14214.17	10,200	15,120	16,400	12,980
G5	C_1262_H_2528_N_506_O_252_	28824.81	14,494	30,570	28,200	24,940
G6	C_2542_H_5888_N_1018_O_508_	58298.21	28,000	54,190	54,000	48,100
G7	C_5102_H_10208_N_2042_O_1020_	116488.71	32,100	98,340	100,200	97,300

Of additional note in the AF4 fractograms is the tendency of the higher-generation dendrimers to show additional secondary peaks at higher elution times. These generally have molecular weights approximately double the parent dendrimer peak and are likely PAMAM dimers, either formed by association in solution or as defects in manufacturing processes, similar to those observed in the HPLC. It is noteworthy here that these peaks are not visible in GPC, and this may be due to the acidic buffer used in the MD-SEC. In acidic solutions, there is more solution phase stability, meaning aggregates are less likely to occur and thus are not seen in GPC. As AF4 separation is purely size-based, it offers an attractive option to analyze dendrimer growth because of further functionalization that causes an increase in size; this is likely to be the case when peptides, proteins, or other polymers are conjugated to the dendrimer surface. This notwithstanding, AF4 method development is more involved than other size separation techniques and thus provides a more complex barrier to entry for routine analysis.

#### Matrix-Assisted Laser Desorption Ionization Time of Flight Mass Spectrometry

The most commonly used mass spectrometry method for the analysis of dendrimers is MALDI.^37,38^ However, single charging of dendrimers is particularly difficult due to the high number of amines, both as primary amines on the surface, and tertiary amines and amides in the core of the dendrimer.^39^

Full mass spectra are available in the Supporting Information (Figures S14–S20). Briefly, G1 is shown as a singly charged species with isotopic distribution, and all other generations show multiply charged peaks. While this is generally a rarity in MALDI spectra, it is not uncommon for PAMAM dendrimers.^40^ For completeness, the resolutions and mass errors for each sample have also been included in the SI (Table S4).

Broadly, for lower generation samples, there is excellent agreement between molecular weights calculated based on idealized molecular formulas and those from MALDI mass spectrometry. As the dendrimer generation increases, larger discrepancies develop between the calculated and measured molecular weights, with the measured molecular weights relatively decreasing. This is likely due to the impurity types previously discussed, including ring-closing between two arms or lack of arms, continuing growth, and becoming more apparent. It should be noted that the reported MALDI masses are for the largest ion peak, and particularly for the higher generation dendrimers, the peaks become broad and lose resolution due to the large range of potential chemical species present. In G1 and G2, the isotopic resolution can be seen between peaks. G1 shows an almost completely pure sample, with very few structural impurity peaks at very low intensity. In G2, multiply charged dendrimers are seen, and more structural impurities are present. For G3, the isotopic resolution between peaks is lost; however, the polymeric nature can be seen, with a distribution of masses present. The mass difference between peaks is 56 Da, corresponding to an ethylene diamine unit, suggesting the presence of dendrimers in the sample, missing certain branching units. This same difference can be seen in the G4 sample again, suggesting there is a mixture of molecules present, of which some are lacking parts of their theoretical structure. For G5–7, the peak-to-peak resolution is also lost, and the peaks seen are a broad distribution of masses, once again suggesting there is a mixture of molecules present. MALDI-TOF MS provides a useful tool for the accurate determination of dendrimer masses and is likely to be the most applicable for observing absolute changes in mass arising from surface functionalization. However, for larger generation dendrimers (G4 and upward), the usefulness diminishes due to the broad peaks observed with no resolution between repeating units. Therefore, larger mass differences, greater than 1000 Da, would be required to observe meaningful changes in the spectra.

#### Small Angle X-ray Scattering

As shown above, due to the relatively small size of PAMAM dendrimers, techniques such as static light scattering (SLS) or DLS can be a challenge to use meaningfully—particularly with lower-generation dendrimers or dendrimers in the presence of larger particles. An alternative to these techniques is SAXS. With laboratory SAXS sources being more readily available from multiple manufacturers and easier to access than synchrotron beamline sources, SAXS is becoming an increasingly more accessible characterization technique. SAXS further has a broad size range available for characterization, typically from 0.5 to 200 nm,^41^ which makes it ideal for all generations of PAMAM that are commercially available. SAXS studies of PAMAM dendrimers have, until recently, been performed only at national synchrotron facilities using complex beamlines, and often only on select generations of PAMAM dendrimers.^42^ Figure 6 shows the scattering curves of PAMAM dendrimers G0–7 collected on our laboratory SAXS instrument, with their associated fits based on a spherical model. Residuals of the fit compared with the experimental data are also available (Figure S23) and show generally good agreement.

**Figure 6:**
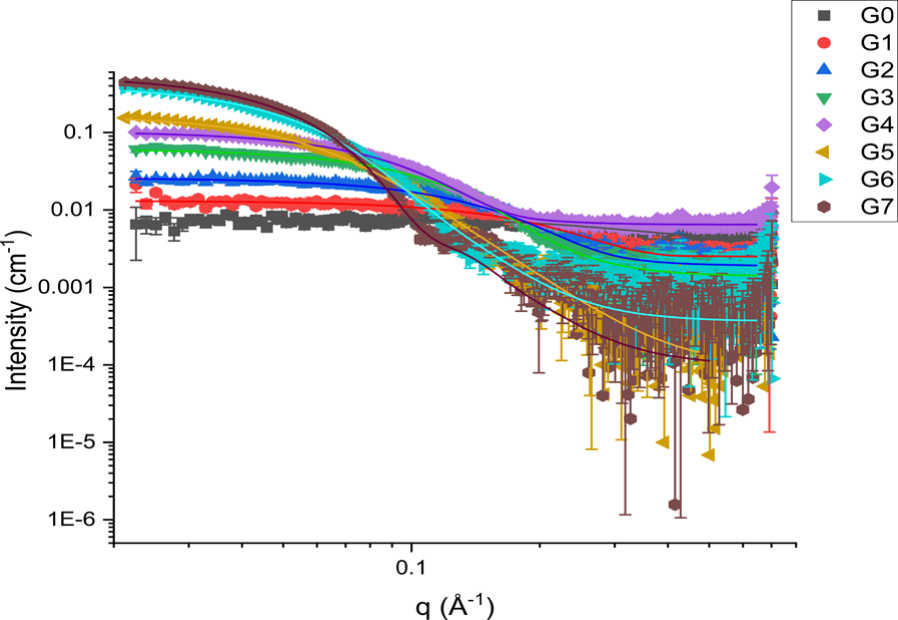
SAXS *q*-plots and associated fits for PAMAM G0–7 dendrimers.

The fits from Figure 6 can be subsequently used to yield size distributions of the PAMAM dendrimers, and these sizes calculated are shown in Table 2, with distributions available in the Supporting Information (Figure S22). It should be noted that due to the relatively high background, the data above 0.2 Å^–1^ possess poor uncertainty and thus are difficult to fit. Further, the high background may be obscuring structure factors contributing to the calculation of dispersity. Additionally, due to the relatively weak scattering seen from the dendrimers, the data in Figure 6 are the result of 4 h of acquisition per sample. This time may be reduced through using a synchrotron beamline; however, acquisition times of 1–6 h were still required in the previous literature examples. This relatively long acquisition time means that SAXS does not provide a useful tool for reaction monitoring in real-time. However, the measurable increases in scattering and size throughout the generations render it a useful tool for confirmation and size determination of products of reactions using dendrimers. Additional analysis opportunities are also present using SAXS, as interparticle interactions can be studied at lower *q*.^42^

**Table 2:** Particle Size Information through Multiple Techniques of PAMAM G0–7

sample	SAXS *R*_g_/nm	SANS *R*_g_/nm	DOSY NMR *R*_h_/nm	DLS *R*_h_/nm	zeta potential/mV	GPC *R*_h_/nm
G0	0.70 ± 0.10		0.61		5.83 ± 0.82	0.73
G1	1.01 ± 0.16	0.41 ± 0.09	0.95		15.22 ± 0.82	1.13
G2	1.22 ± 0.36	0.83 ± 0.06	1.45		17.67 ± 0.97	1.61
G3	1.52 ± 0.55	1.18 ± 0.08	1.87		15.02 ± 0.99	2.14
G4	2.20 ± 0.49	1.38 ± 0.05	2.99	1.81 ± 0.42	15.30 ± 0.96	2.94
G5	2.22 ± 1.46	2.29 ± 0.04	3.69	2.53 ± 0.60	13.83 ± 0.96	3.67
G6	3.40 ± 1.12	2.82 ± 0.05	5.48	3.03 ± 0.66	17.33 ± 0.98	4.39
G7	4.09 ± 0.77	3.12 ± 0.05	5.95	4.48 ± 0.71	16.55 ± 0.95	5.30

#### Small-Angle Neutron Scattering

The clear alternative for comparable data to SAXS is SANS. While SANS facilities are generally more difficult to access, due to the low number of high-flux neutron facilities worldwide, a better contrast can be achieved in SANS through the use of deuterium.^43,44^ This can be performed by building the polymer with deuterated monomers^45^; however, this is often prohibitively expensive. An alternative method for this involves running the experiment in a deuterated solvent. The benefits in contrast between SAXS and SANS for dendrimers can be seen by calculating theoretical scattering length density (SLD)^46^ values, which show a difference of 5.083 × 10^–6^/Å^2^ for the system of PAMAM G4 in D_2_O for SANS, compared to just 1.865 × 10^–6^/Å^2^ for the same system in SAXS.

Limited SANS characterization data have been previously reported for dendrimers, with scattering profiles available for G3–7 at various concentrations^47^ and simulated profiles available for further generations.^48^ However, there are currently no data available on the experimental scattering profiles of most PAMAM dendrimers or a comparison between multiple techniques. Figure 7 shows the SANS scattering profiles of PAMAM dendrimers G0–7, along with their respective fits.^49^

**Figure 7:**
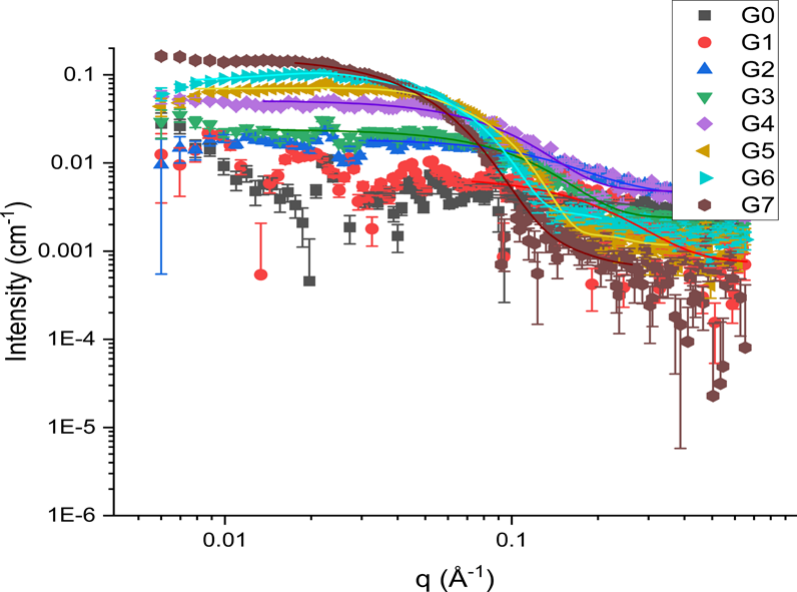
SANS *q*-plots and associated fits for PAMAM G0–7 dendrimers.

As with the SAXS data, there is a clear increase in scattering as a function of dendrimer generation due to the larger size. However, unlike the SAXS data, the uncertainty at higher q is reduced, allowing for more accurate data fitting for this region and therefore more certainty around the dispersity. Additionally, the lower q available in SANS allows us to see the interparticle interaction that is present and indeed was predicted by the zeta-potentials. The calculated sizes are shown in Table 2, and size distributions are available in the Supporting Information (Figure S24). Residuals of the fit compared to the experimental data are also available (Figure S26) and show generally good agreement.

The fast acquisition times afforded by SANS make it an ideal solution for studying the reaction kinetics and the accompanying size changes in real-time.

#### Comparisons between Techniques

Throughout this work, several techniques have been used to characterize commercial PAMAM dendrimers, particularly those relating to size and mass; each of these has its associated drawbacks and benefits, and a comparison between the masses calculated can be seen in Table 1. When comparing these techniques to the calculated molecular weights from idealized structures, we can see that MALDI, GPC, and AF4 broadly show good agreement.

For G0, most of these techniques, which are geared toward large molecule analysis, struggle to provide meaningful data, and more traditional small molecule approaches should be used, such as LC-MS.

For low generations (G1–3), the agreement between the molecular weight calculated by all three techniques and the theoretical values is extremely close, except for G1 via AF4, which suffers from being outside the effective calibration window. This suggests that the structural impurities present do not have a noticeable effect on the average molecular weight. Indeed, this can be seen by looking at the HPLC chromatograms, where the major peak dominates the separation. The types of impurities present in these dendrimers, therefore, are likely intramolecular ring closing, where the ring closure happens close to the surface of the dendrimer, and therefore, the molecular weight only changes from the idealized structure by a few Daltons. With these samples, the choice of instrument is specific to the use case. Where accurate masses are required or mass differences are small, for example, small molecule conjugation to the dendrimer surface, MALDI is likely to yield the most information, although it comes at a higher cost and is more challenging to acquire data than chromatographic techniques. Meanwhile, if mass differences are large or there is a broad range of sizes to separate, GPC or AF4 is likely more appropriate. For a faster, rough measurement of molecular weight, MaDDOSY also provides a valuable tool, especially as the same sample used for NMR analysis can be used.

As the dendrimer gets larger (G4–7), the molecular weight changes from the ideal are more pronounced, and this is seen across MALDI, GPC, and AF4. Of note is that MALDI data have masses significantly lower than expected and even lower than the other two techniques. This may be a true reading of the molecular weight; however, it could also be due to the difficulty of ionizing larger species, and so more signal is seen for lower molecular weight dendrimer defects than higher. The lower-than-expected molecular weights are seen across analytical techniques, not only with MALDI, and so it is likely that the majority of the dendrimers at higher generations have defects present that reduce the effective molecular weight, such as intramolecular cyclizations that occur at lower generations and then propagate throughout the synthesis.

Across the techniques at higher generations, the masses given do show deviation between each other, although in each case, with the exception of MaDDOSY, this deviation is within expected values. GPC was performed using light scattering with single-point calibration using PEO as a standard, and while this is typical for polymeric systems, dendrimers do indeed take on different architectures when compared to linear polymers in solution, so, as such, the value may be improved by using other calibrants if available. For the purposes of reaction monitoring, this could even be the use of native dendrimers as calibrants. In comparison, AF4 shows the closest agreement with the theoretical values, and this is likely due to the use of BSA as the calibrant in this system, as previously discussed. MaDDOSY, however, fails to give realistic values at higher generations, and this is likely due to the universal nature of the calibration inherent in the MaDDOSY technique. As the authors in the original work describe, a separate calibration, using more appropriate calibrants, may be required for more accurate results.

As previously mentioned, MALDI as a tool loses usefulness from generation 5 upward, producing distributions that look similar to GPC chromatograms or AF4 fractograms. Therefore, for generations 5 and upward, we recommend that molecular weight determination be performed using GPC or AF4, due to their lower instrument cost and ease of use. For G1–4, MALDI can provide useful information around functionalization and mass distributions, and should be used in tandem with other techniques to study size.

Alongside the mass information obtained through these techniques, particle sizing has also been investigated throughout this study. A comparison of the sizes obtained is shown in Table 2. While SAXS and SANS measure the radius of gyration (*R*_g_), DLS and DOSY NMR measure the hydrodynamic radius (*R*_h_). *R*_h_ is defined as the radius of an equivalent hard sphere, which diffuses at the same rate as the molecule under investigation, whereas *R*_g_ is the mass-weighted average distance from the core of a molecule to each mass element of the molecule.^50–52^ In practice, this means that *R*_h_ is always larger than *R*_g_ for sphere-like particles, and indeed for a globular protein, this relationship is roughly approximated to *R*_g_/*R*_h_ ≈ 0.775.^53^ The errors associated with the SAXS, SANS, and DLS data are in each case the standard deviations calculated from fitting.

It can be seen from these data that, as expected, the size in all techniques increases with increasing dendrimer generation. Additionally, there is excellent agreement between the hydrodynamic radii measured by DOSY NMR and multidetector GPC, with results from DOSY NMR similar to results previously reported.^54^ The data obtained from DLS is considerably less consistent with respect to the other techniques used, and this could be attributed to either the use of lower pH than in other techniques, causing electrostatic effects, resulting in a smaller effective radius,^26^ or as previously mentioned, the practicality of running DLS measurements on particularly small particles, where aggregates can dominate the scattering signal. For this reason, more complex techniques are generally required to further understand the size of the whole suite of PAMAM dendrimers.

As techniques, SAXS and SANS offer a unique insight into nanoparticles such as dendrimers, by allowing significantly more in-depth probing of the chosen size range. When the acquired SAXS data are examined, the trends previously described are once again present, with increasing size as a function of dendrimer generation and the relationship between *R*_g_ and *R*_h_ described above being clearly present. The values calculated from SANS are, however, generally considerably smaller than those from SAXS, and this lower measured radius is likely due to there being solvent molecules between the arms of the dendrimer. This reduces the contrast between the solvent and the analyte, decreasing the measured radius, as has been previously shown.^55^ Additionally, it is noteworthy that generally broader distributions are seen in the SANS data than in the SAXS data, which can be determined through fitting the lower q regions that are available from the SANS experiment.

Once again, depending on the information required, different techniques stand out as the technique of choice. For hydrodynamic radii, DOSY NMR and GPC provide the best options for the entire range of dendrimers; however, both suffer from their inability to provide useful information on the size distribution of dendrimers. For larger dendrimers (G4–7), DLS then becomes a more powerful technique, where the scattering is strong enough to give reliable data on the sizes as well as the distribution of those sizes. If *R*_g_ data are instead required, static scattering experiments are required. As all sizes of dendrimer are below 10 nm, SLS is not possible, and therefore, the most accessible technique is SAXS. Overall, however, the sizes reported for both techniques are in agreement with the literature but provided herein as a full data set.^56,57^

To compare the analytical techniques used further, shape factors (*R*_g_/*R*_h_) have been calculated between all techniques used. These are detailed in Table 3. Typically, the value of 0.775 described above describes a sphere of uniformly distributed mass. Values lower than this, generally around 0.6, represent a system which has a denser core and looser shell, and values greater than this, around 1, represent a particle where more of the mass is located on the shell of the system. Values above 1 are considered no longer spherical objects, with random coils having values of up to 1.5.^58^

**Table 3:** Shape Factors *R*_g_ /*R*_h_ for PAMAM Samples Using Different Techniques, Anomalous Results Marked with *

sample	SAXS/GPC	SAXS/DOSY	SAXS/DLS	SANS/GPC	SANS/DOSY	SANS/DLS
G0	0.959	1.148*				
G1	0.894	1.063*		0.363	0.432	
G2	0.758	0.841		0.516	0.572	
G3	0.710	0.813		0.551	0.631	
G4	0.748	0.736	1.215*	0.469	0.462	0.762
G5	0.605	0.602	0.877	0.624	0.621	0.905
G6	0.774	0.620	1.122*	0.642	0.515	0.931
G7	0.772	0.687	0.913	0.589	0.524	0.696

Aside from three anomalous results (marked with a * in Table 3), all samples, regardless of the technique used, show a spherical shape factor. The shape factors for the SAXS/DLS samples show the most deviation, and this is likely due to a combination of the poor scattering contrast afforded by SAXS and the need to fit a relatively small region of the curve, combined with the inherent insensitivity DLS has toward smaller particles. The low-generation SAXS/DOSY samples also show anomalous shape factors, and this is likely due to the Stokes–Einstein relation not being a sufficiently good model to calculate hydrodynamic radii at sub-1 nm, whereas above this size, it works adequately. In the cases where SAXS was used for the values of *R*_g,_ it is generally seen that for G0–2, the shape factor is close to 1, indicating the majority of the mass is within the shell of the molecule. This result is not particularly unexpected, given that in lower-generation dendrimers the core is sterically hindered; therefore, the arms of the shell can account for a larger proportion of the mass. From G2 onward, the results using SAXS/GPC show that the particle is spherical, with broadly uniformly distributed mass. With the SAXS/DOSY data, it is seen that G2 and G3 have spherical shapes with more mass toward the shell, G4 has uniformly distributed mass, and G5–7 have more mass in the core than the shell. These results are also interesting, as the dendrimer increases in size, most of the mass shifts toward the core; this suggests that the pendant surface groups are less constrained and may help with surface functionalization. Where SANS is used for *R*_g_ determination, the shape factors are universally much smaller; this follows from the much smaller radii measured during this experiment in comparison to SAXS.

Overall, the scope of analytical techniques used in this study shows that a broad range of techniques can be applied to PAMAM dendrimers; however, the use cases for each technique vary. For utility, each analytical technique is outlined, in addition to its utility for various applications, in Table 4. Alongside this is the typical cost per hour of instrument time in USD for a research institution.^59^ It should be noted that these costs for industrial applications are generally much higher, and for SANS time in particular, the cost to academic users is usually free via a merit-based proposal, and as such, the cost here is an industry estimate per hour.

**Table 4:** Comparison of Analytical Techniques Based on Use for Dendrimers, Cost, and Applications

technique	applicable generations	cost/hour of instrument time (USD)	applications
NMR (80 MHz)	G0–7	25	routine analysis and functionalization monitoring
DOSY (80 MHz)	G1–7	25	conjugation monitoring and indirect calculation of *R*_h_
IR	G0–7	9	routine analysis, limited functionalization monitoring
UV–vis	G0–7	15	reaction monitoring
DLS	G4–7	25	calculation of *R*_h_ and size distribution
ζ-potential	G0–7	25	reaction monitoring, estimates of nanoparticle stability
HPLC	G2–7	18	QC analysis, limited reaction monitoring
GPC	G1–7	24	estimation of molecular weight and molecular weight distribution, calculation of *R*_h_ if using light scattering
AF4	G1–7	73	QC analysis, estimation of molecular weight and molecular weight distribution, calculation of *R*_h_ and *R*_g_ if using MALS
MALDI-MS	G1–4	54	direct measurement of molecular weight, limited functionalization verification
SAXS (Lab Source)	G0–7	47	direct measurement of *R*_g_, fitting to estimate size distributions, limited functionalization verification
SANS	G1–7	363*	direct measurement of *R*_g_, fitting to estimate size distributions, functionalization verification, reaction monitoring

## Conclusions

PAMAM dendrimers present many novel opportunities in the chemical space, with particular interest in offering enhancements to drug portfolios and new technologies for formulation science. However, the challenges associated with their synthesis and analysis can present significant obstacles to their uptake for these applications.

Using pre-existing knowledge alongside novel methods, a suite of analytical techniques has been applied to globally characterize G0–7 of PAMAM dendrimers. Broadly, these techniques have fallen into two categories: those that yield chemical information and those that provide size information. Chemically, techniques including IR and UV–vis spectroscopy, NMR, and zeta-potential provide useful insights into the surface functionality of the native dendrimers. This provides a particular opportunity for reaction monitoring applications, as the primary amine surface functionality, which is often exploited for further functionalization, can be simply monitored through observing changes in surface charge or chemical environment.

Multiple techniques are available for the assessment of the size of PAMAM dendrimers. While mass spectrometry provided an important bridge between chemical and size information, we have found that, as with previous literature, it generally fails to provide useful information at higher dendrimer generations, with MALDI data only giving an approximate idea of molecular weight with no peak-to-peak resolution. As a more accessible technique, GPC also provides molecular weight information that demonstrates excellent agreement with MALDI data. Additional molecular weight data have also been provided by AF4, which again shows excellent agreement with the other techniques. In practice, any of these techniques is acceptable for the collection of molecular weight data, and often, the availability of equipment and size of dendrimer should be used to guide the choice of technique.

To provide a greater overview regarding the particle size, which is crucial for the functionality and properties of the particle for many applications, multiple sizing techniques have been tested. Both calibrated sizing techniques and fitting techniques have been used, with DOSY NMR, GPC, and DLS able to provide valuable information about hydrodynamic radius. The work here shows that while DLS may be the easiest technique to access, it does not provide reproducible size data for PAMAM dendrimers and is highly sensitive to the presence of larger particles, including aggregates. DOSY NMR and GPC, meanwhile, show excellent agreement with each other and are often reasonably accessible for use. DOSY has the additional benefit of providing chemical data, which can be used to assess, for example, the uptake of a ligand during a conjugation reaction.

SAXS and SANS have also been used to provide information on the radius of gyration of the various PAMAM dendrimers, showing a clear increase in size as a function of dendrimer generation. In these cases, it has been shown that SAXS generally proves a better technique than SANS, partially due to the higher availability of instruments, with the inclusion of laboratory sources, but also because the structure of PAMAM allows solvent molecules to enter the outer shell, causing a problem for the contrast achieved in SANS, resulting in smaller measured radii. SANS remains a useful option for PAMAM dendrimers due to improved contrast with solvent, allowing significantly faster measurement times, which may be crucial for reaction monitoring purposes.

By comparing the *R*_g_ and *R*_h_ measurements acquired, shape factors have been calculated, which clearly show that for lower generation dendrimers, a more star-like structure is adopted, which transforms into a nearly uniform sphere at higher generations.

While the investigations conducted in this work provide a broad overview of the analytical techniques available to characterize PAMAM dendrimers, they are by no means exhaustive; more in-depth mass spectrometry techniques, including FT-ICR, could provide a greater in-depth understanding of the PAMAM microstructure, allowing a greater understanding of the impurities present. Additionally, the size and shape of PAMAM dendrimers are known to be highly solvent-dependent, and so more work needs to focus on how the physicochemical properties discussed in this work are affected in various solvent systems, particularly those that are application-specific. More work is also needed on the characterization of the impurities, which can provide challenges to PAMAMs' commercial use and may involve the use of preparative separation techniques to allow full chemical characterization. Greater attention should also be focused on reaction monitoring using these techniques in order to optimize reaction processes for commercially relevant samples.

## Supplementary Material



## Notes

Table S1 - Peakintegrals from 1H NMR of PAMAM G0-7 in methanol-d4; Table S2 - Diffusionconstants, hydrodynamic radii and MaDDOSY molecular weights as measured by 1HDOSY in methanol-d4 (500 MHz); Table S3 - Dendrimer purity by mathematical peakarea at 210 nm; Table S4 - Resolution and mass error for PAMAM samples analyzedby MALDI-TOF-MS; Table S5 - Physico-chemical parameters of PAMAM dendrimersobtained by triple-detection GPC; Table S6 - Scattering length densities (SLDs)­calculated for D2O and PAMAM G4 in both SAXS and SANS; Figure S1 - 1H COSY NMRspectrum for PAMAM G1; Figure S2 - 1H-13C HSQC of PAMAM G1 in methanol-d4; FigureS3 - 13C NMR Spectra for PAMAM generations G0-2 in methanol-d4; Figure S4 - 1HDOSY of PAMAM G4 in methanol-d4; Figure S5 - Hydrodynamic radii and MaDDOSYmolecular weights for PAMAM G0-7; Figure S6 - Infrared Spectrum of PAMAM G0-7in methanol; Figure S7 - Infrared Spectrum of PAMAM G0-7 (Methanol Subtracted);Figure S8 - UV-Vis spectra for PAMAM G0-7; Figure S9 - Correlation functionsfor PAMAM G0-7; Figure S10 - Volume weighted size distributions for PAMAM G0-7;Figure S11 - Normalized volume weighted size distributions for PAMAM G4-7; FigureS12- Normalized zeta-potential measurements for PAMAM G0-7 in 0.1M pH 3.0citrate buffer; Figure S13 - HPLC chromatograms of PAMAM G0-7; Figure S14 -MALDI-TOF-MS for PAMAM G1; Figure S15 - MALDI TOF MS for PAMAM G2; Figure S16 -MALDI-TOF-MS for PAMAM G3; Figure S17 - MALDI-TOF-MS for PAMAM G4; Figure S18 -MALDI-TOF-MS for PAMAM G5; Figure S19 - MALDI-TOF-MS for PAMAM G6; Figure S20 -MALDI-TOF-MS for PAMAM G7; Figure S21 - Full AF4 Fractograms for PAMAM G1-7; FigureS22 - Volume weighted size distributions obtained from SAXS; Figure S23 -Residuals from SAXS fit vs Raw Data; Figure S24 - Waterfall plot of q vsintensity acquired from SAXS for PAMAM G0-7; Figure S25 - Volume weighted sizedistributions obtained from SANS; Figure S26 - Residuals from SANS fit vs RawData; Figure S27 - Waterfall plot of q vs intensity acquired from SANS forPAMAM G0-7 (PDF)

## Abbreviations

AF4: asymmetric field flow field fractionation
ATR: attenuated total reflection
BSA: bovine serum albumin
COSY: correlation spectroscopy
DLS: dynamic light scattering
HPLC: high-performance liquid chromatography
DOSY: diffusion-ordered spectroscopy
DREAM: distribution evolution with self-adaptive randomized subspace sampling
EDA: ethylene diamine
GNAT: general NMR analysis toolbox
GPC: gel permeation chromatography
HSQC: heteronuclear single quantum correlation
ILL: Institut Laue-Langevin
IR: infrared
MaDDOSY: mass determination diffusion ordered spectroscopy
MALDI: matrix-assisted laser desorption ionization
MALS: multiangle light scattering
MS: mass spectrometry
NMR: nuclear magnetic resonance
PAMAM: poly­(amido amine)
PDA: photodiode array
PEG/PEO: poly­(ethylene glycol)/poly­(ethylene oxide)
PSS: polymer standards service
Rg: radius of gyration
Rh: hydrodynamic radius
RI: refractive index
SLS: static light scattering
SANS: small-angle neutron scattering
SAXS: small-angle X-ray scattering
SEC: size exclusion chromatography
TFA: trifluoroacetic acid
TOF: time of flight
UV–vis: ultraviolet visible
