# Supplementary material for: Global Characterization of Commercial Generation 0–7 Poly(amidoamine) Dendrimers: Challenges and Opportunities for Analysis
Source: ACS Omega. 2025 Oct 1;10(40):47582–95. doi: 10.1021/acsomega.5c07827 (PMC12529379; doi:10.1021/acsomega.5c07827)
Supplement: Supplementary file 1 [file ao5c07827_si_001.pdf]

SUPPORTING INFORMATION FOR: GLOBAL CHARACTERIZATION OF COMMERCIAL GENERATION 0 TO 7 POLY(AMIDOAMINE) (PAMAM) DENDRIMERS: CHALLENGES AND OPPORTUNITIES FOR ANALYSIS

Owen Tooley[a], William Pointer[a], Rowan Radmall[a], Steve Huband[b], James Town[c], Anne Martel[d], Patricia Monteiro[e] *, Thomas Floyd[f] *, Paul Wilson[a]* and Daniel Lester[c]*

[a] Department of Chemistry, University of Warwick, Coventry, CV4 7AL, United Kingdom

[b] X-Ray Diffraction RTP, University of Warwick, Coventry, CV4 7AL, United Kingdom

[c] Polymer Characterization RTP, University of Warwick, Coventry, CV4 7AL, United Kingdom

[d] Institut Laue-Langevin (ILL), 71 Av. des Martyrs, 38000 Grenoble, France

[e] Advanced Drug Delivery, Pharmaceutical Sciences, R&D, AstraZeneca, Cambridge, UK

[f] Advanced Drug Delivery, Pharmaceutical Sciences, R&D, AstraZeneca, Macclesfield, UK

NUCLEAR MAGNETIC RESONANCE SPECTROSCOPY

Complete raw data is available upon request. Peak integrals for Figure 1 of the main manuscript are given in table 1.

Table S1 - Peak integrals from ^{1}H NMR of PAMAM G0-7 in methanol-d4.

	a	b	c	d	e	f
G0	2.19	1	1.01	2.15	2.13	2.07
G1	2.01	1	0.04	1.30	1.92	1.99
G2	1.73	1	0.09	0.98	1.63	1.61
G3	1.98	1	0.03	1.00	2.05	1.68
G4	1.96	1	0.02	0.90	1.96	2.07
G5	2.02	1	-	0.98	2.00	1.85
G6	1.96	1	-	0.90	1.93	1.91
G7	1.98	1	-	0.99	1.84	1.84

In order to assign the NMR spectra, ^{1}H COSY (Figure 1) and 1H-^{13}C HSQC (Figure 2) experiments were performed. For completeness representative ^{1}H COSY and ^{1}H-^{13}C spectra are provided for PAMAM G1.

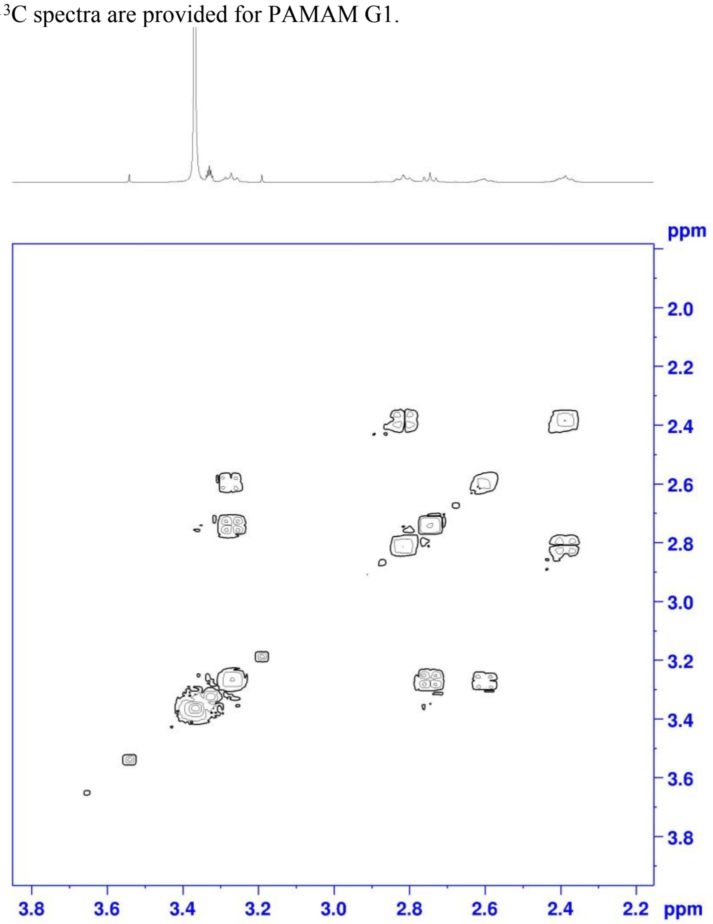

Figure S1 - ^{1}H COSY NMR spectrum for PAMAM G1

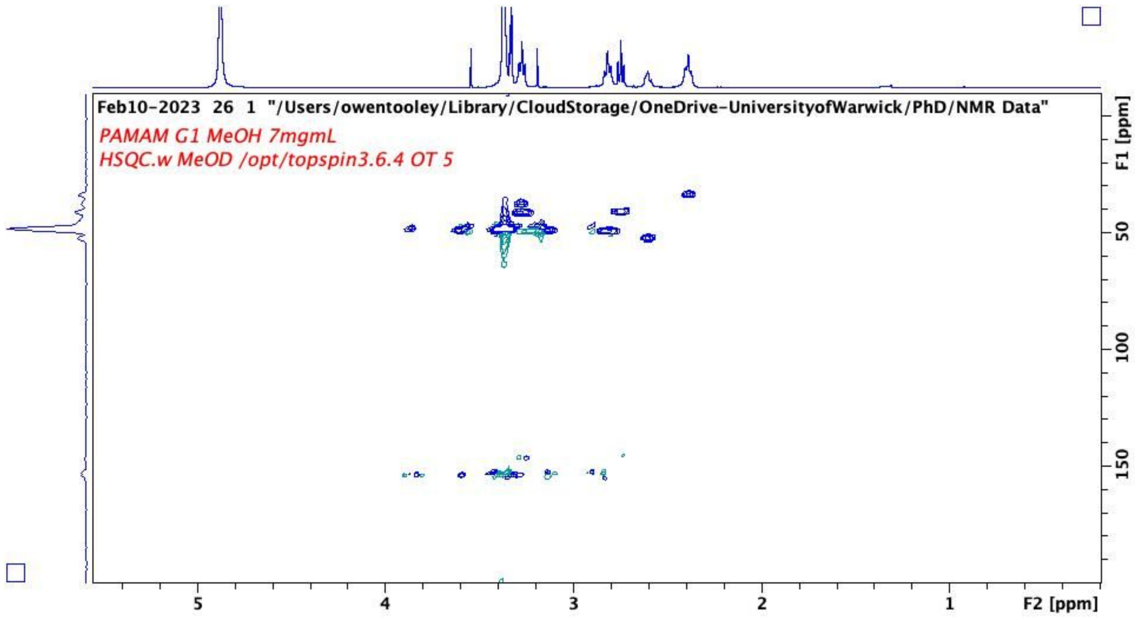

Figure S2 - ^{1}H-^{13}C HSQC of PAMAM G1 in methanol-d4

Representative ^{13}C NMR data are shown in Figure 2 for Generations 0 – 2.

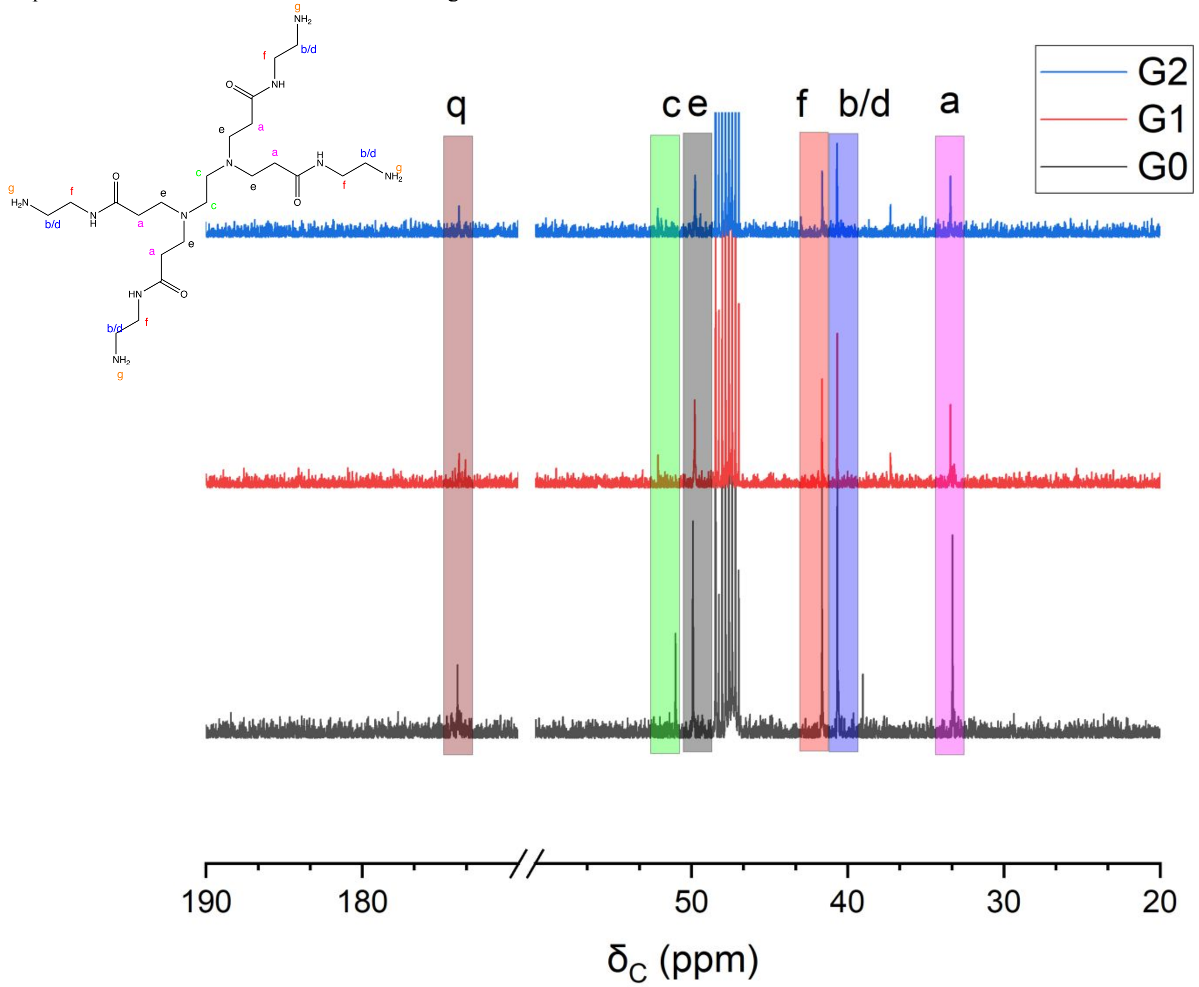

Figure S3 - ^{13}C NMR Spectra for PAMAM generations G0-2 in methanol-d4.

A representative DOSY spectrum (Figure 4) for PAMAM G4 and the diffusion constants from the DOSY experiments, alongside their calculated hydrodynamic radii and MaDDOSY molecular weights (Table 2), are provided.

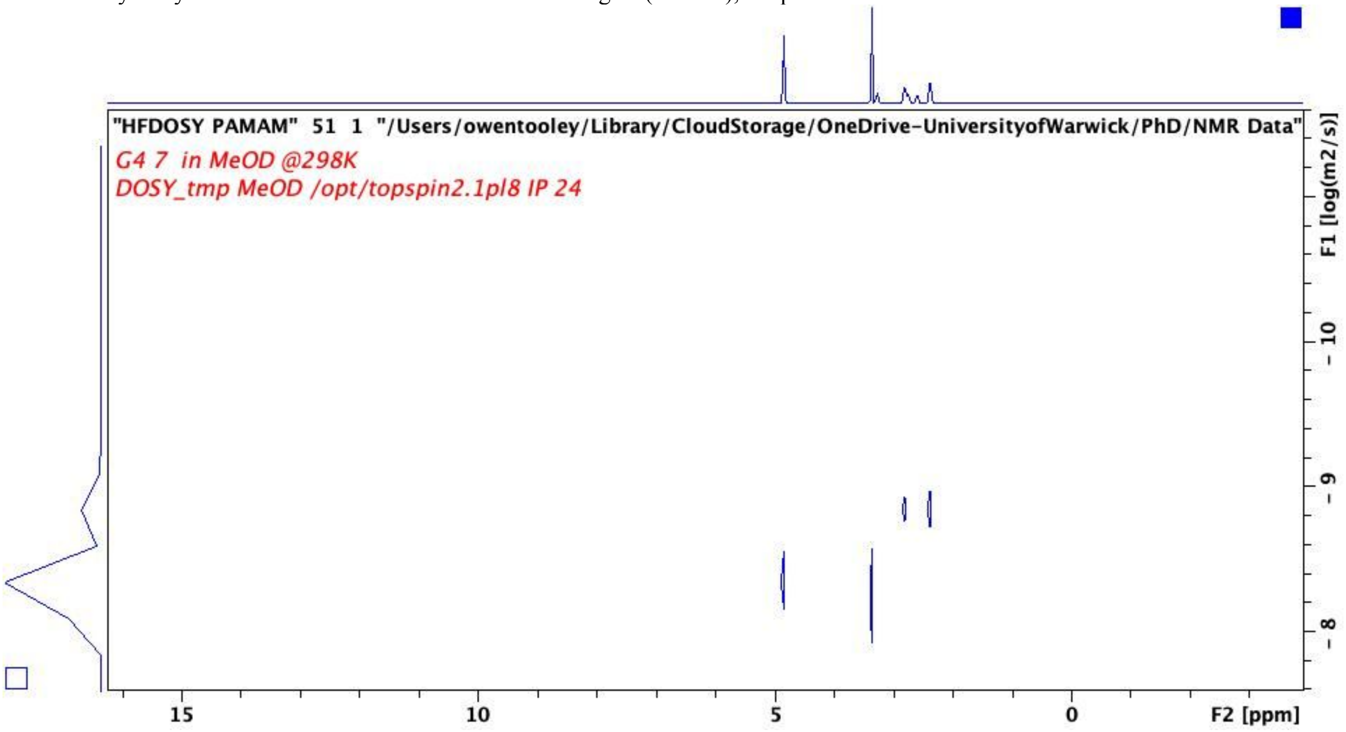

Figure S4 - ^{1}H DOSY of PAMAM G4 in methanol-d4.

The hydrodynamic radii were calculated using the Stokes-Einstein equation (Equation 1), with a solvent viscosity methanol-d4 of 0.597 mPa S and a temperature of 298 K.

Equation 1 - Stokes-Einstein Equation, D is the diffusion constant, k_B the Boltzmann constant, T the temperature, η the bulk viscosity of the solvent and R_H the hydrodynamic radius

$$D = \frac{k_B T}{6\pi\eta R_H}$$

Table S2 - Diffusion constants, hydrodynamic radii and MaDDOSY molecular weights as measured by 1H DOSY in methanol-d4 (500 MHz)

	Diffusion constant ($x10^{-10}$ m^2 s^{-1})	Hydrodynamic Radius (nm)	MaDDOSY Molecular Weight (g mol^{-1})
G0	6.92	0.61	700
G1	4.44	0.95	1500
G2	2.91	1.45	3000
G3	2.26	1.87	4600
G4	1.41	2.99	10200
G5	1.14	3.69	14494
G6	0.77	5.48	28000
G7	0.71	5.95	32100

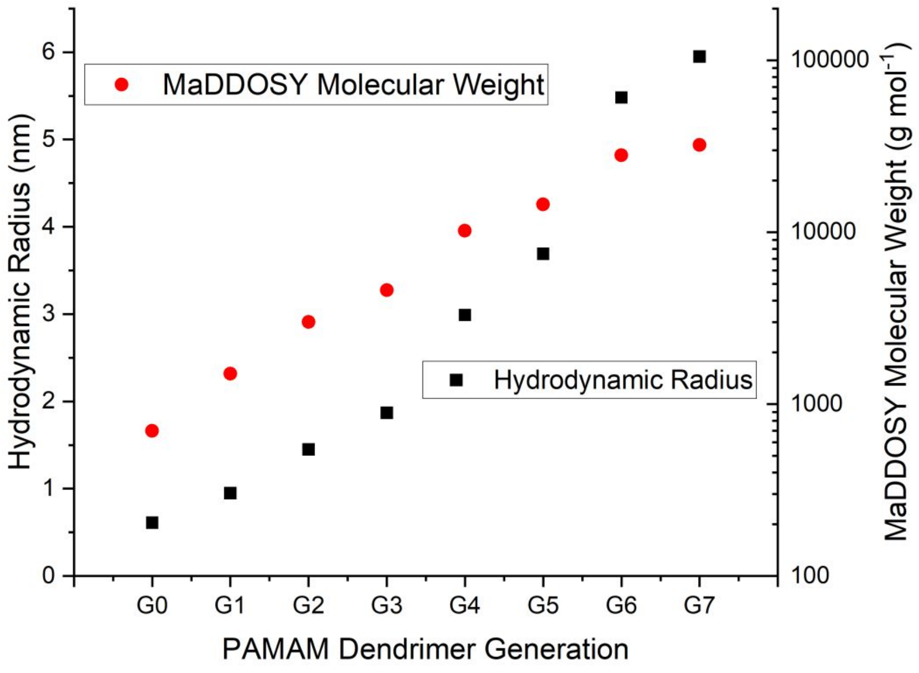

Figure S5 - Hydrodynamic radii and MaDDOSY molecular weights for PAMAM G0-7

INFRARED SPECTROSCOPY

The infrared spectra of PAMAM G0-7 are provided both in methanol (Figure 6) and with a background subtraction of methanol (Figure 7).

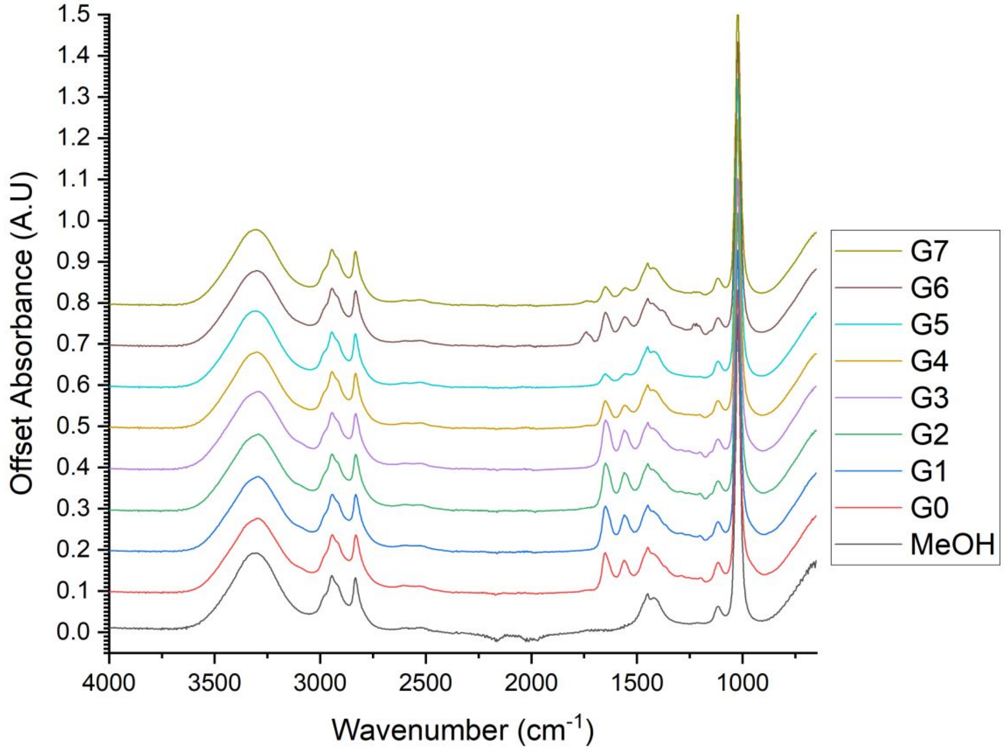

Figure S6 - Infrared Spectrum of PAMAM G0-7 in methanol.

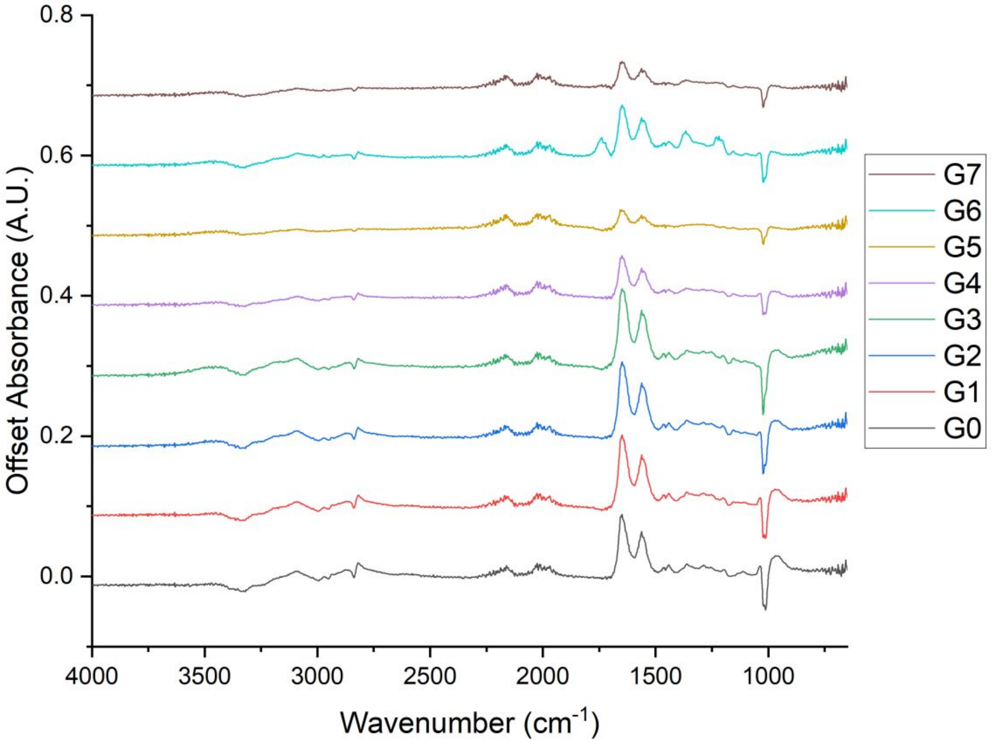

Figure S7 - Infrared Spectrum of PAMAM G0-7 (Methanol Subtracted)

ULTRAVIOLET-VISIBLE SPECTROSCOPY

The UV-Vis spectra of PAMAM G0-7 are provided (Figure 8) , it should be noted the spectrum for G7 is too concentrated for this detector, however, the concentration has been kept the same for all 8 samples to allow direct comparison. Data above 500 nm has been omitted as the molar extinction coefficient for all samples above 500 nm was 0.

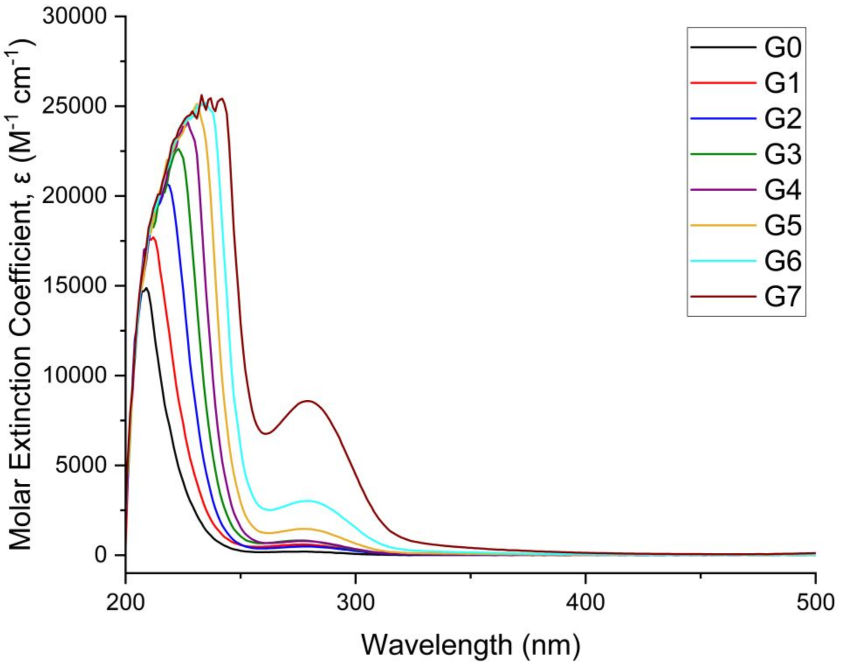

Figure S8 - UV-Vis spectra for PAMAM G0-7.

DYNAMIC LIGHT SCATTERING (DLS) AND ZETA POTENTIAL

The DLS correlation functions are provided for PAMAM G0-7 (Figure 9), G3 is excluded due to no reproducible data being achieved. It is also noteworthy that G0-2 are dominated by larger particles and therefore the data here is likely to be unreliable, as can be seen by the correlation function data and so, for these reasons, the data for G0-3 have been omitted from the normalized data. The corresponding volume weighted size distributions are provided (Figure 10) as well as normalized volume weighted size distributions for G4-7 (Figure 11).

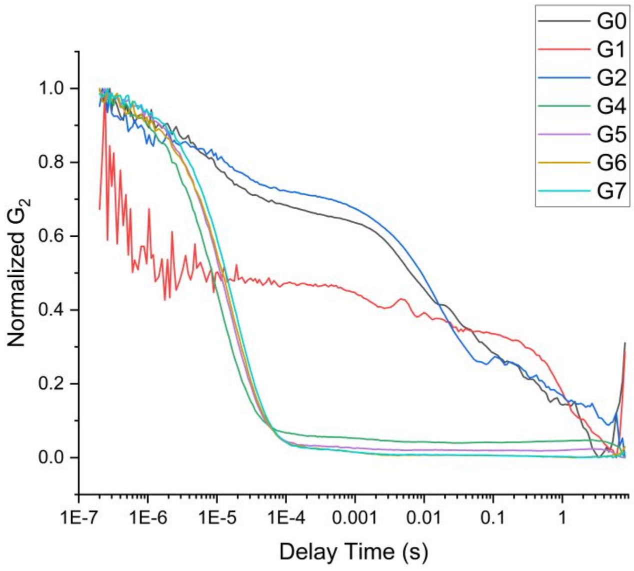

Figure S9 - Correlation functions for PAMAM G0-7.

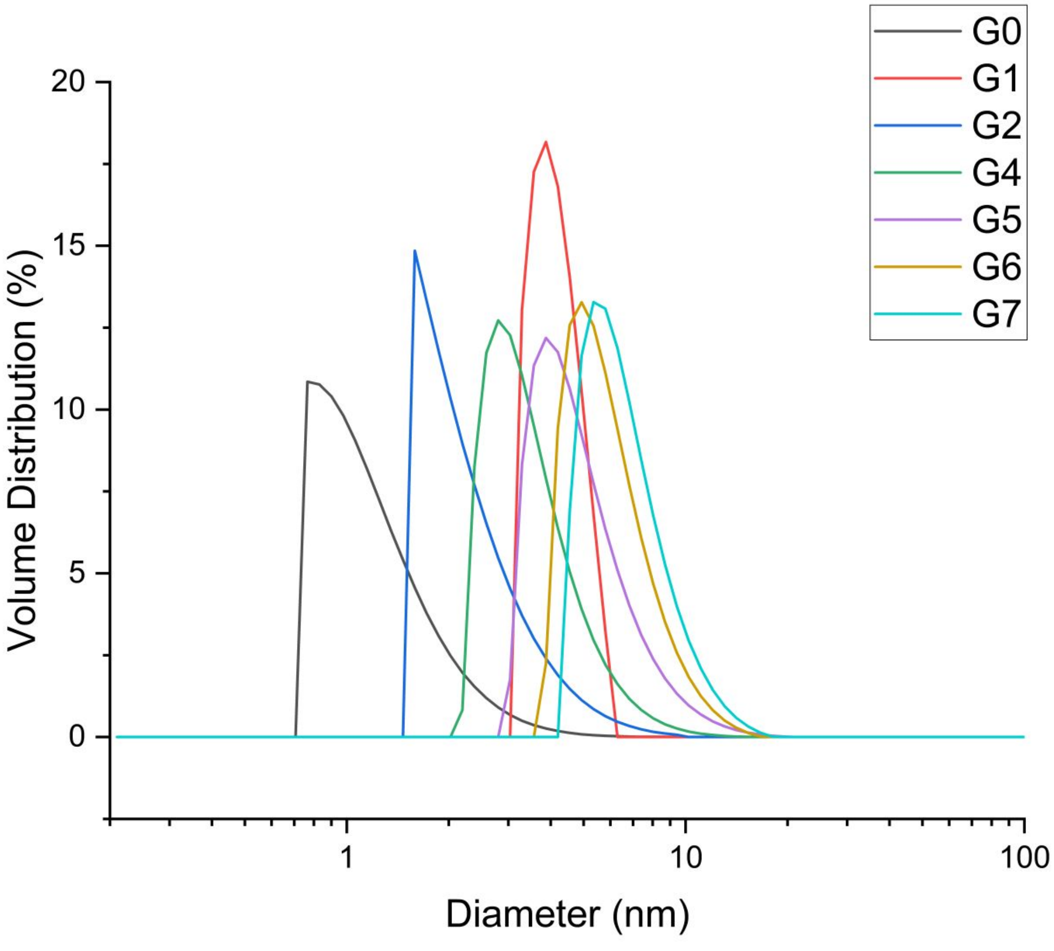

Figure S10 - Volume weighted size distributions for PAMAM G0-7.

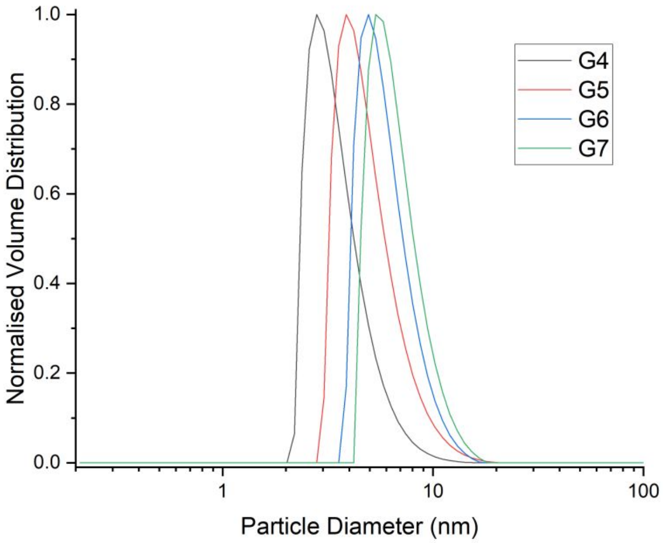

Figure S11 - Normalized volume weighted size distributions for PAMAM G4-7.

The normalized zeta-potential measurement distributions for PAMAM G0-7 in 0.1M pH 3.0 citrate buffer are provided (Figure 12).

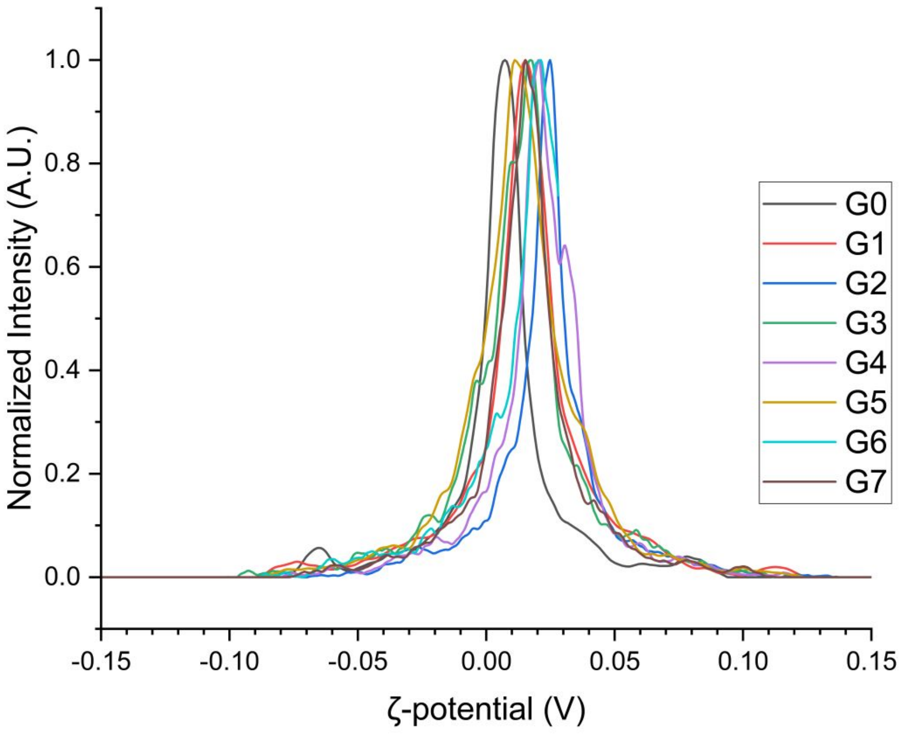

Figure S12- Normalized zeta-potential measurements for PAMAM G0-7 in 0.1M pH 3.0 citrate buffer.

HIGH PERFORMANCE LIQUID CHROMATOGRAPHY

Full HPLC chromatograms, alongside the enlarged region of interest, are shown (Figure 13). Percentage purities by mathematical area at 210 nm are given (Table 3).

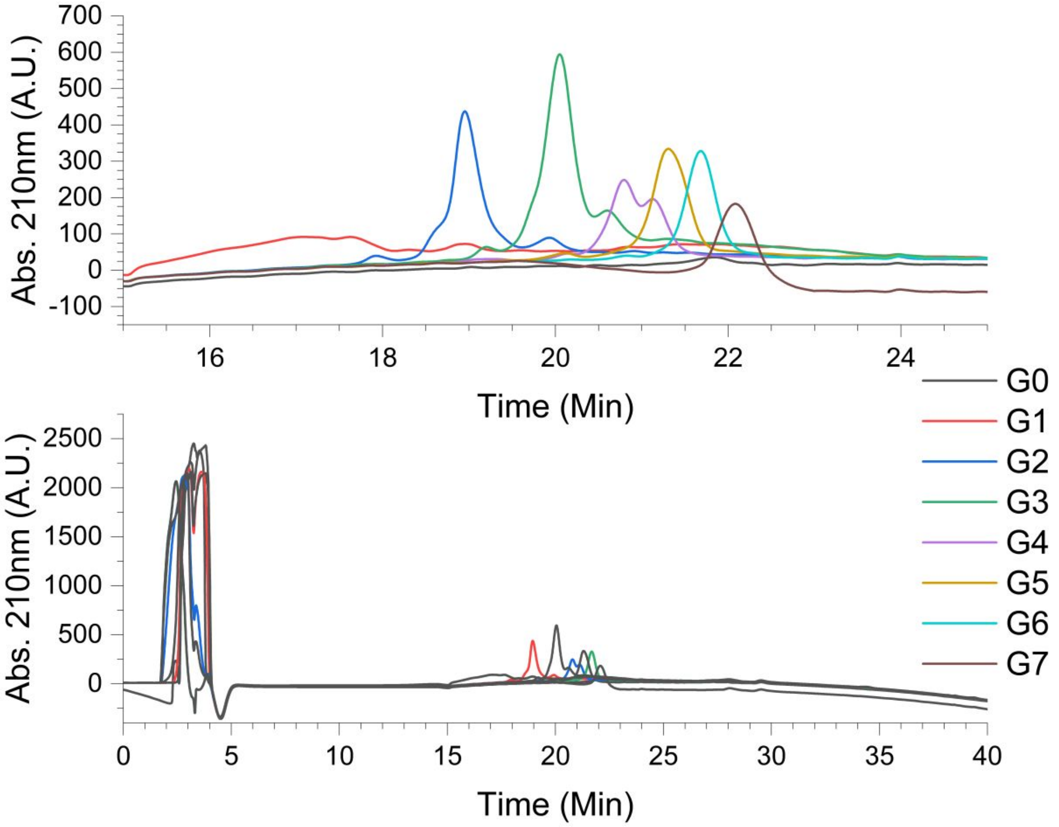

Figure S13 - HPLC chromatograms of PAMAM G0-7

Table S3 - Dendrimer purity by mathematical peak area at 210 nm.

Dendrimer	Purity (%)
G0	Not measurable
G1	Not measurable
G2	84.96
G3	87.91
G4	91.10
G5	90.24
G6	95.32
G7	95.54

MATRIX ASSISTED LASER DESORPTION IONIZATION TIME OF FLIGHT MASS SPECTROMETRY (MALDI-TOF-MS)

The MALDI mass spectrographs for PAMAM G1-7 are provided (Figures 14-20). G0 was not analyzed as its theoretical mass is too small for MALDI-TOF-MS and would likely be obscured by matrix clustering. In each case the spectra have been baseline subtracted and smoothed

where required. The found masses in each case can be seen in the main manuscript, and the resolution of each of the calibrants and mass error of each sample are given (Table 4). The errors here are unusually high for most polymeric systems, suggesting that pure dendrimers are not present.

Table S4 - Resolution and mass error for PAMAM samples analyzed by MALDI-TOF-MS.

Sample	Resolution[45]	Mass Error (ppm)[46]
G1	14292	16190.5
G2	14292	6516.8
G3	1134	10676.2
G4	108	-86826.7
G5	68	-134773.1
G6	68	-174931.8
G7	1.3	-164725.9

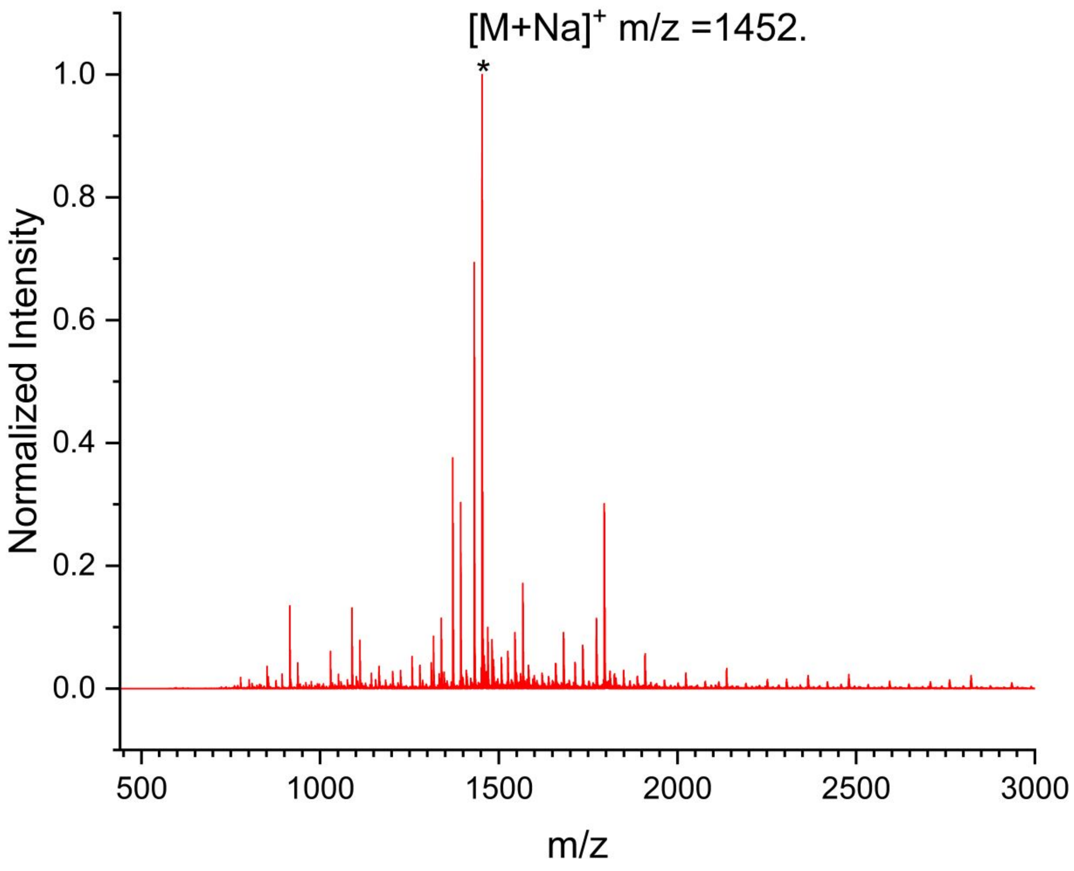

Figure S14 - MALDI-TOF-MS for PAMAM G1.

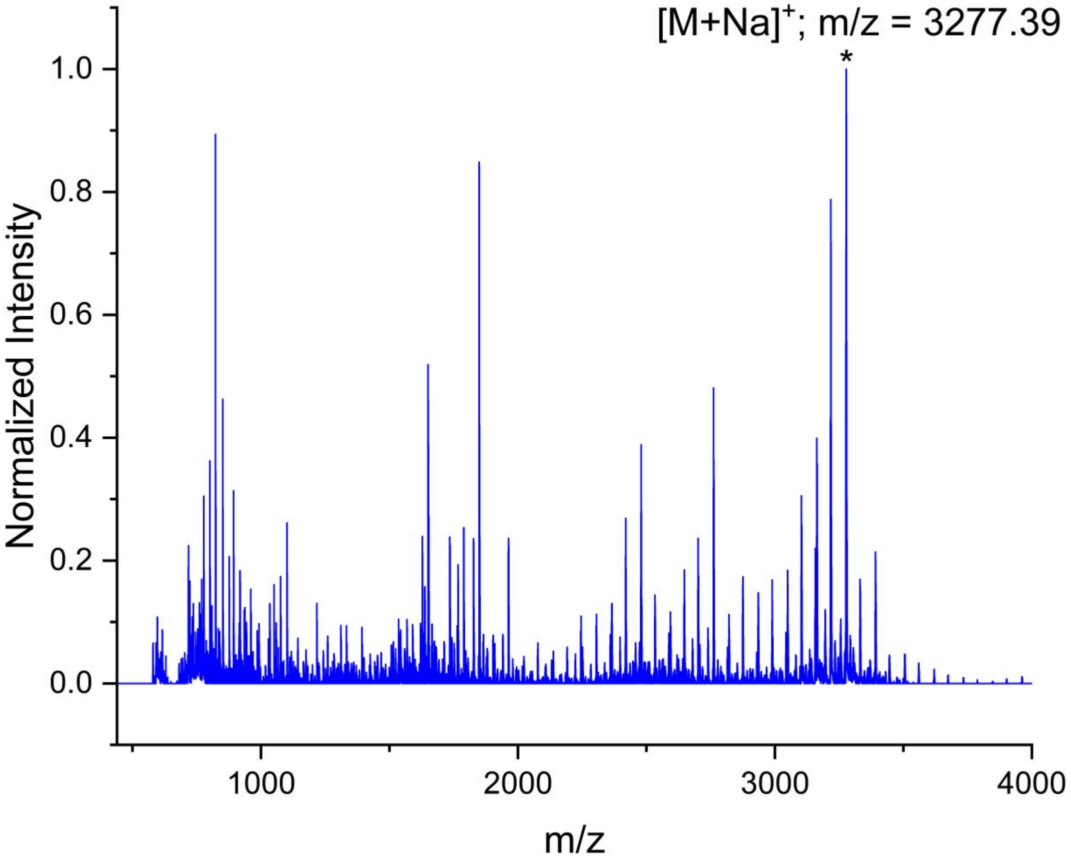

Figure S15 - MALDI TOF MS for PAMAM G2.

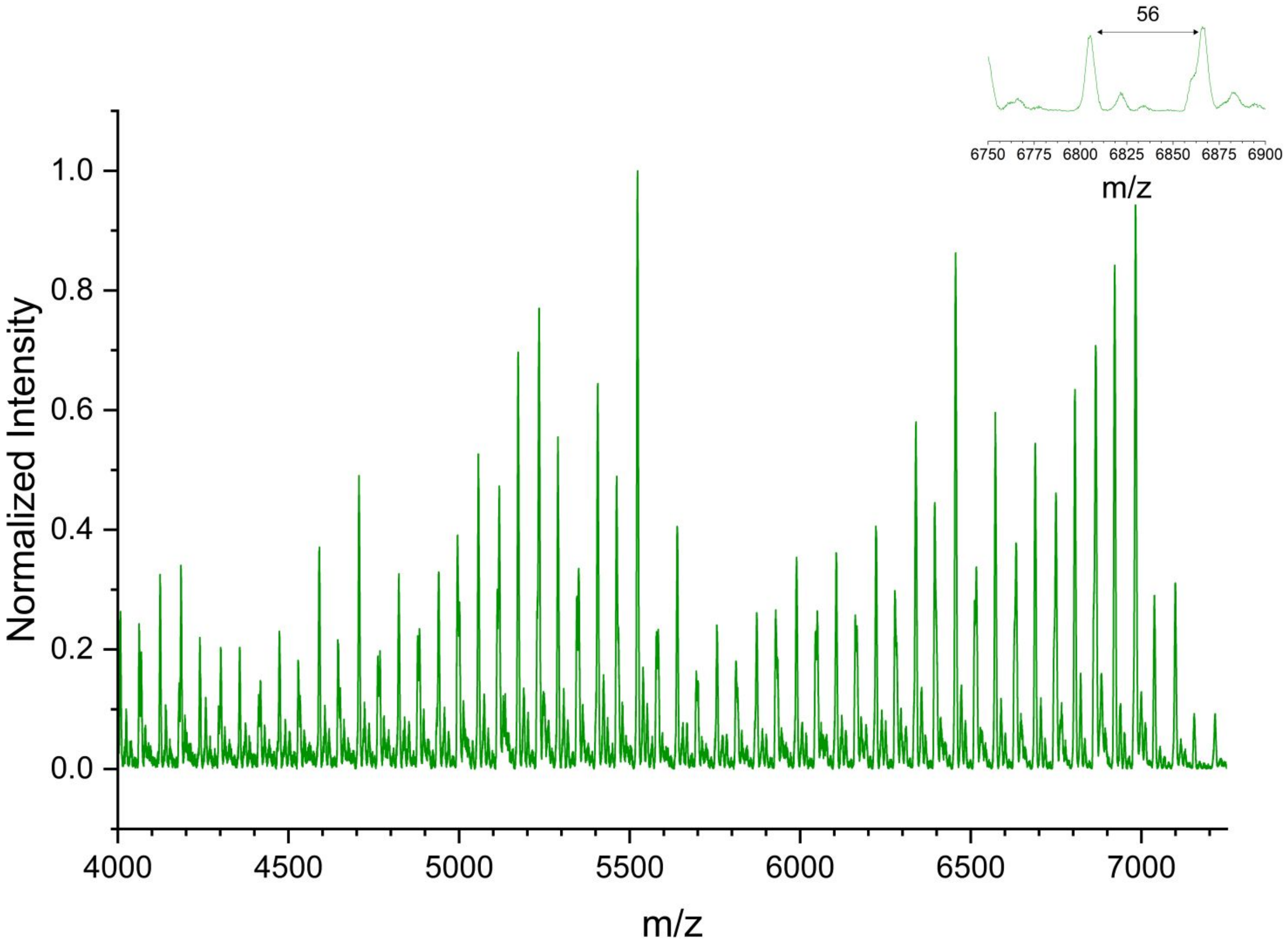

Figure S16 - MALDI-TOF-MS for PAMAM G3.

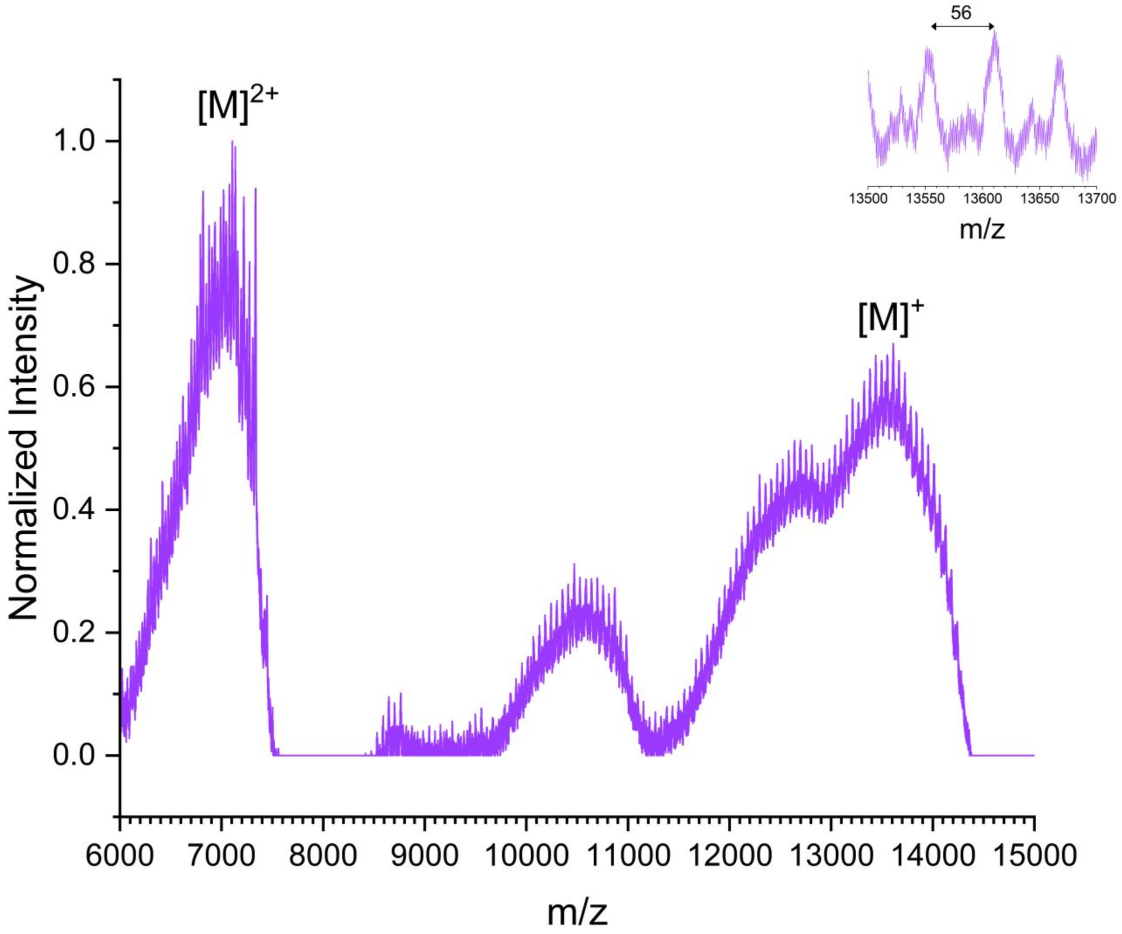

Figure S17 - MALDI-TOF-MS for PAMAM G4.

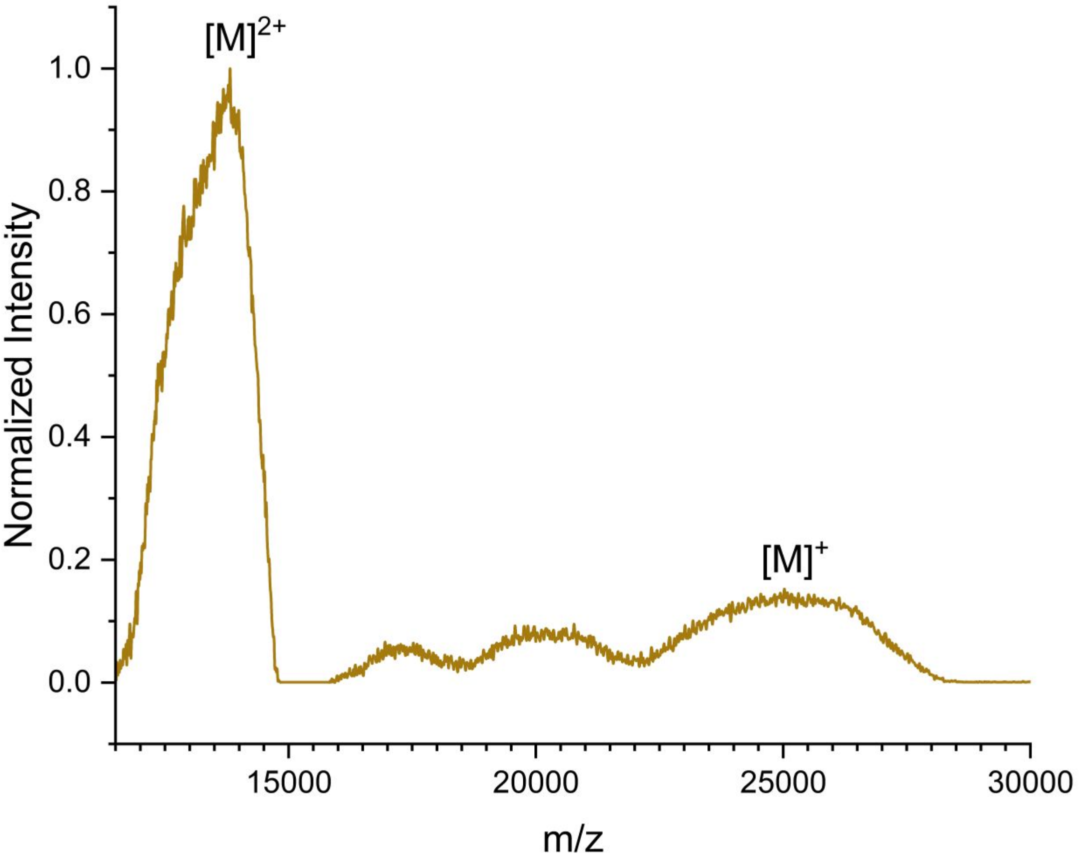

Figure S18 - MALDI-TOF-MS for PAMAM G5.

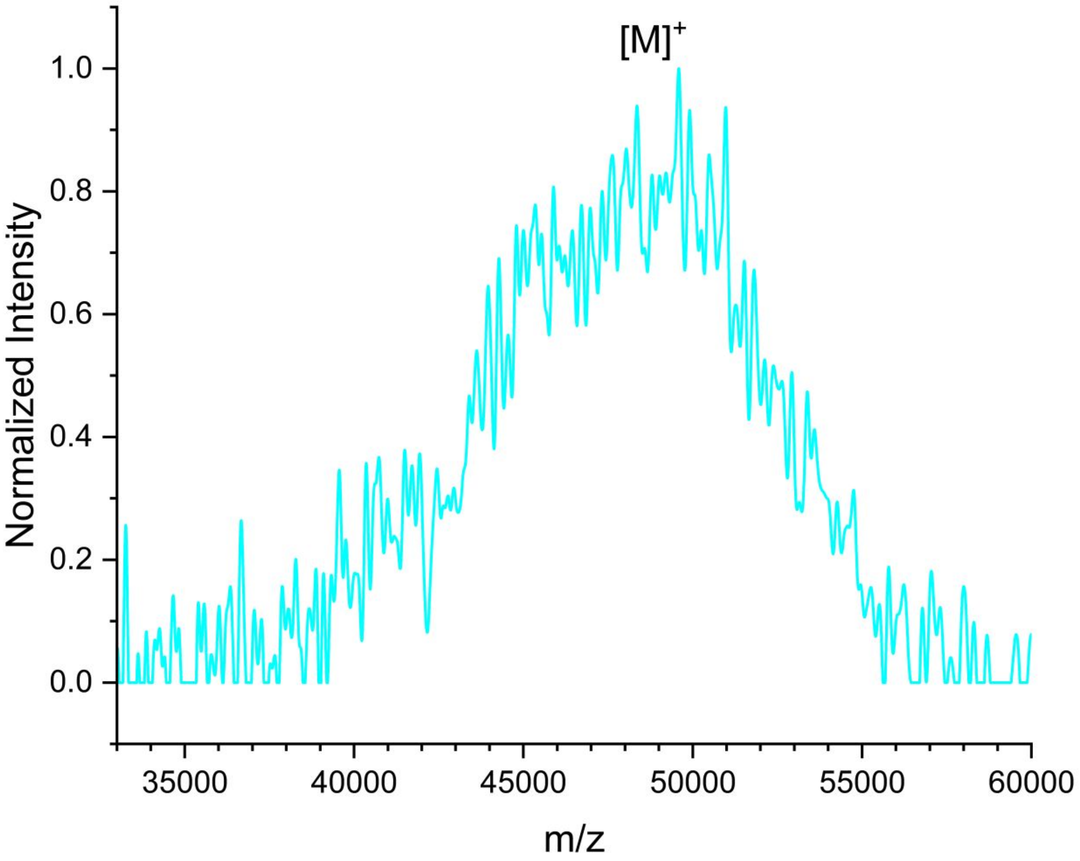

Figure S19 - MALDI-TOF-MS for PAMAM G6.

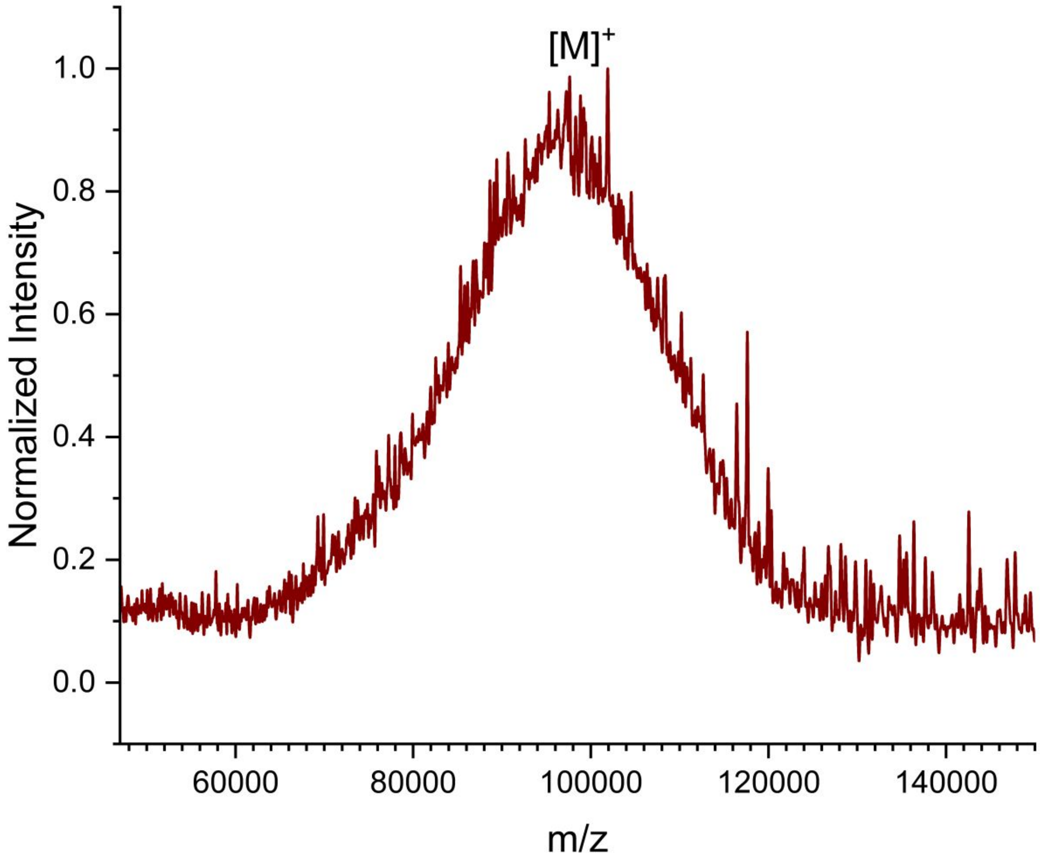

Figure S20 - MALDI-TOF-MS for PAMAM G7.

GEL PERMEATION CHROMATOGRAPHY (GPC)

The physico-chemical parameters of PAMAM G0-7 as determined by triple detection GPC are given (Table 5).

Table S5 - Physico-chemical parameters of PAMAM dendrimers obtained by triple-detection GPC.

Sample	Intrinsic Viscosity /. dL g^{-1}	dn/dc
G0	0.0384	0.232
G1	0.0598	0.236
G2	0.0739	0.236
G3	0.0926	0.258
G4	0.1017	0.236
G5	0.0985	0.229
G6	0.0958	0.241
G7	0.0970	0.248

ASYMMETRIC FIELD FLOW FIELD FRACTIONATION (AF4)

The full AF4 Fractograms are provided (Figure 21)

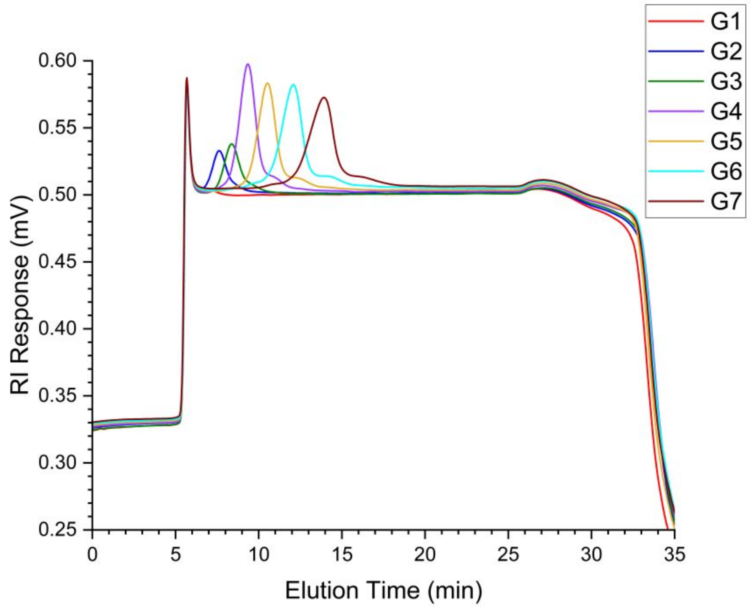

Figure S21 - Full AF4 Fractograms for PAMAM G1-7

SMALL ANGLE X-RAY SCATTERING (SAXS)

The volume weighted size distributions as obtained from SAXS are provided (Figure 22), alongside the residuals from the fit against the raw data (Figure 23), the SAXS q plots are also reproduced without error bars, for clarity (Figure 24).

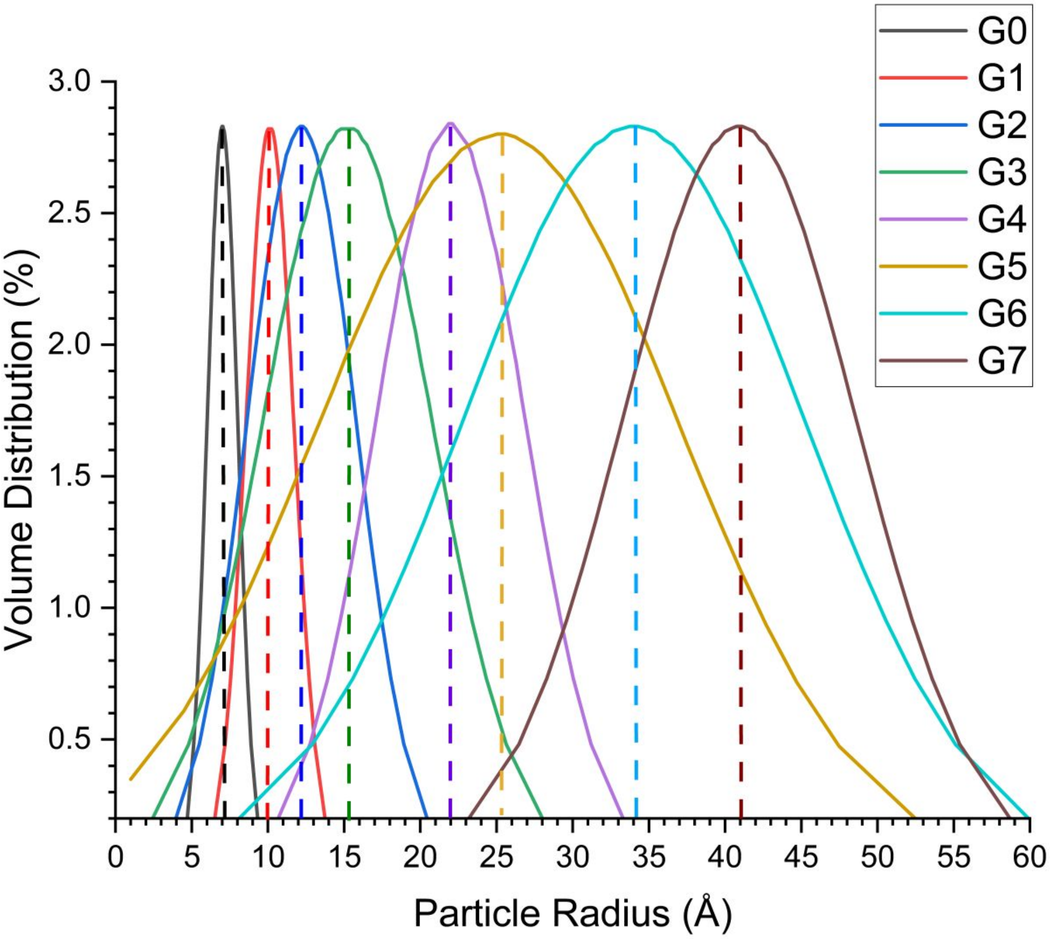

Figure S22 - Volume weighted size distributions obtained from SAXS.

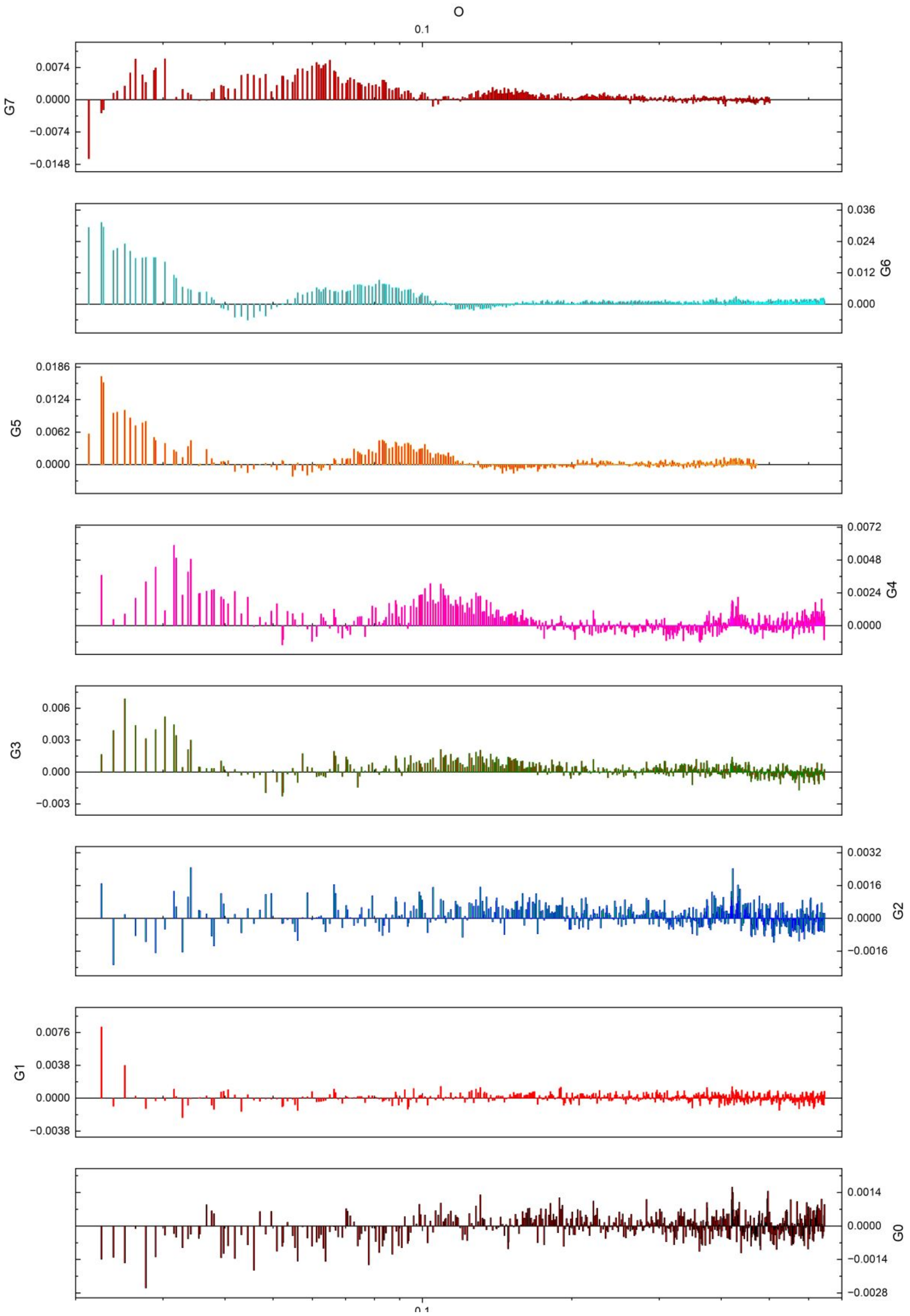

Figure S23 - Residuals from SAXS fit vs Raw Data

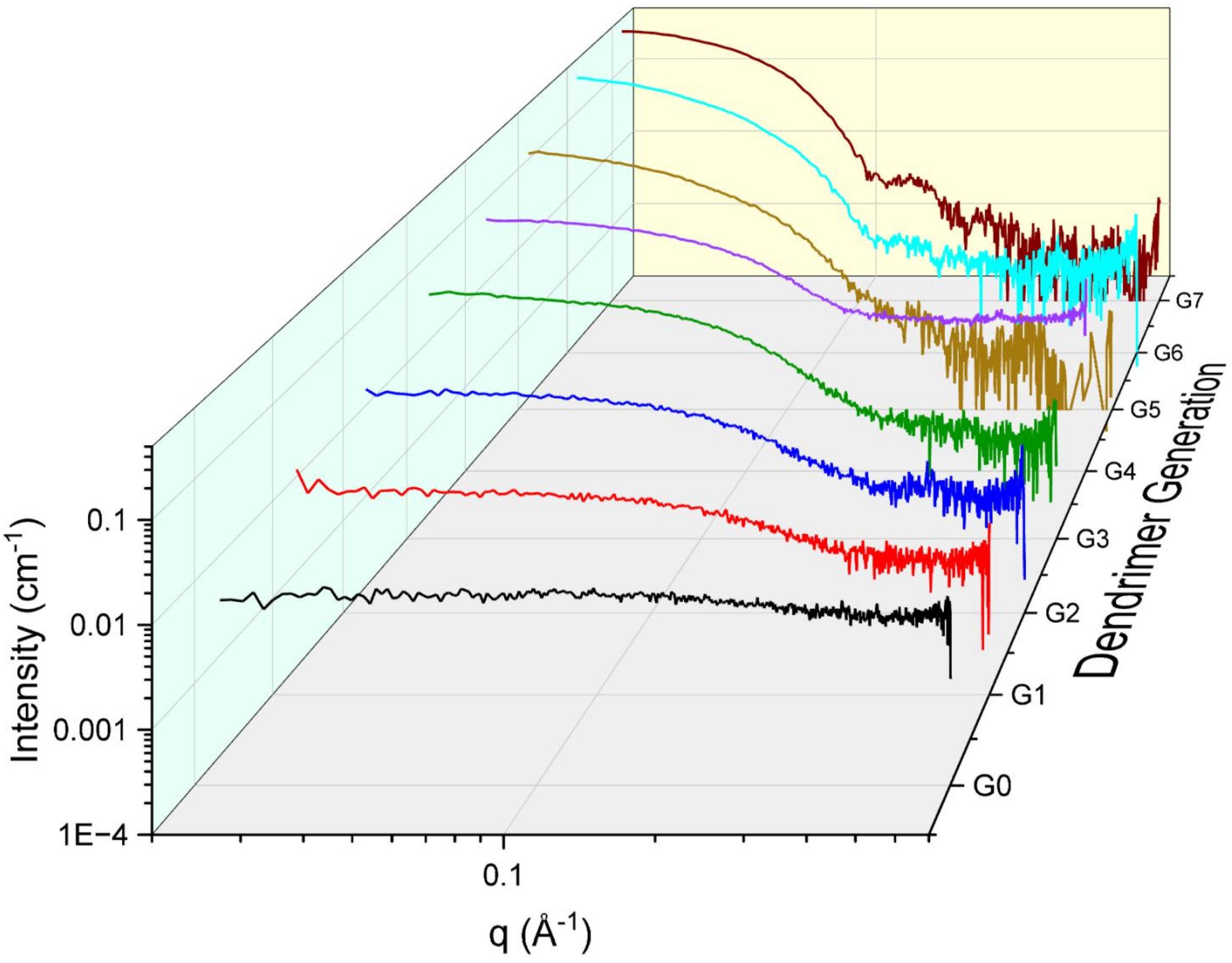

Figure S24 - Waterfall plot of q vs intensity acquired from SAXS for PAMAM G0-7

SMALL ANGLE NEUTRON SCATTERING (SANS)

The calculated scattering length densities of D_2O and PAMAM G4 are provided (Table 6) to show the contrast enhancement afforded by using SANS. The volume weighted size distributions obtained from SANS are provided (Figure 25). Additionally provided are the residuals from the fit against the raw data (Figure 26), the SAXS q plots are also reproduced without error bars, for clarity (Figure 27).

Table S6 - Scattering length densities (SLDs) calculated for D_2O and PAMAM G4 in both SAXS and SANS

	D_2O SLD (10^{-6}/Å^2)	PAMAM G4 SLD (10^{-6}/Å^2)
X-Ray (Cu Kα)	9.455	11.32
Neutron (6 Å)	6.393	1.31

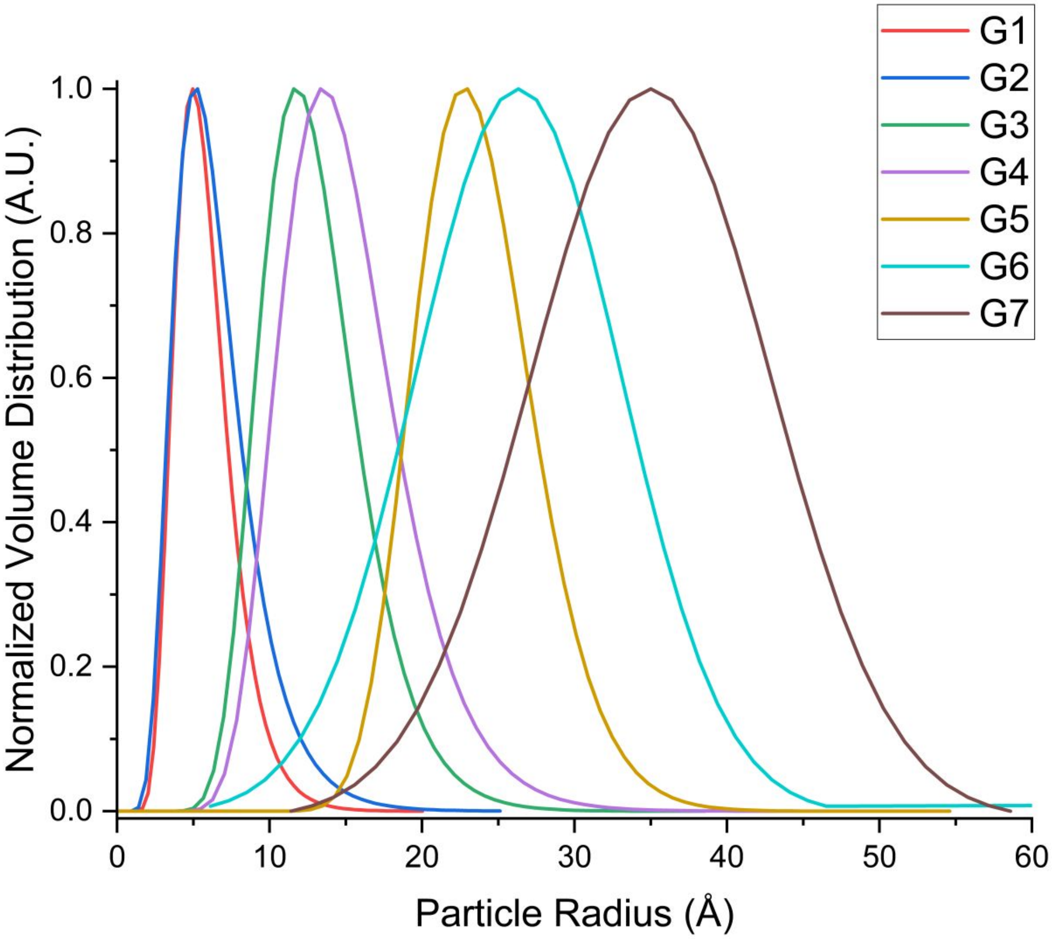

Figure S25 - Volume weighted size distributions obtained from SANS.

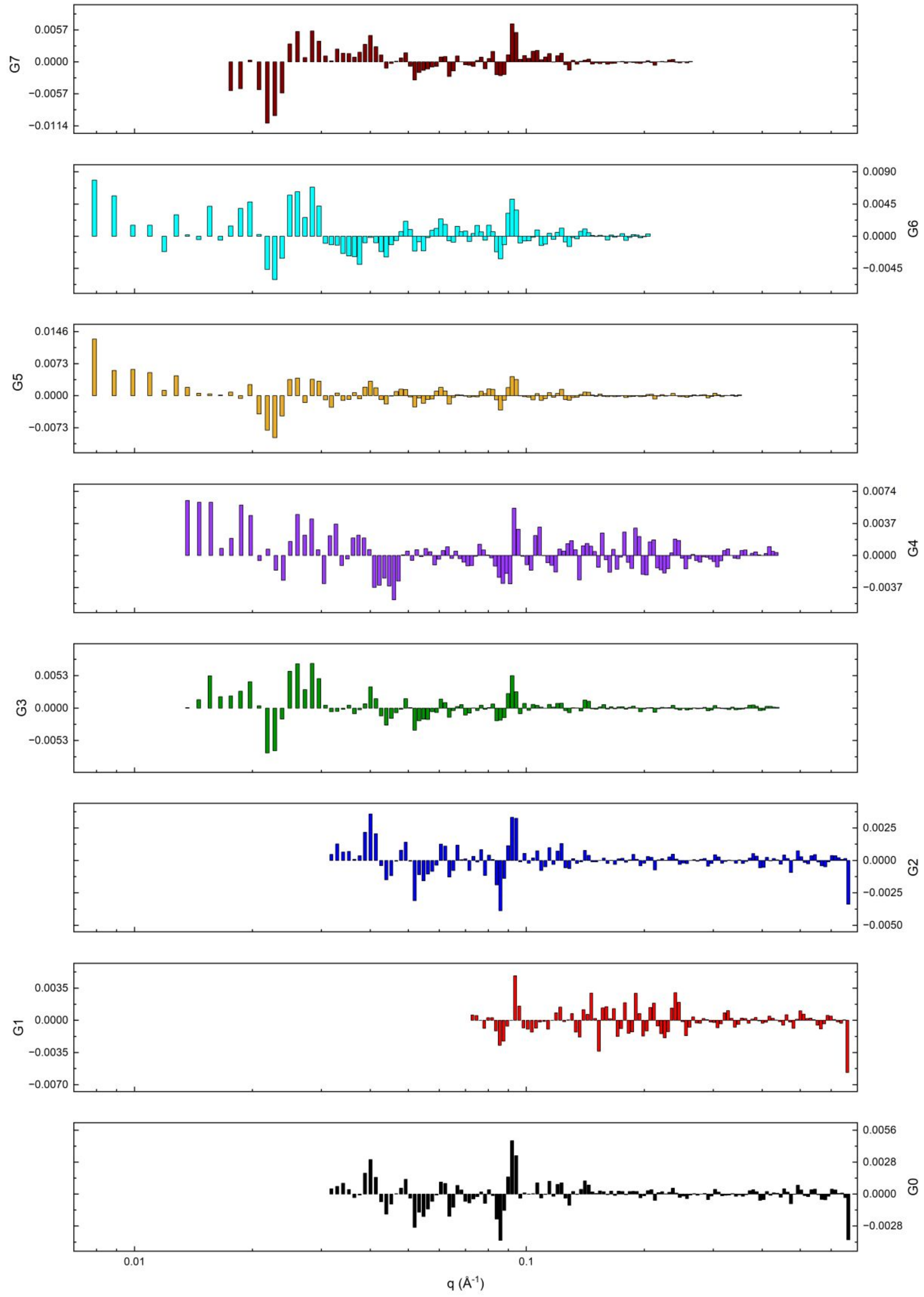

Figure S26 - Residuals from SANS fit vs Raw Data

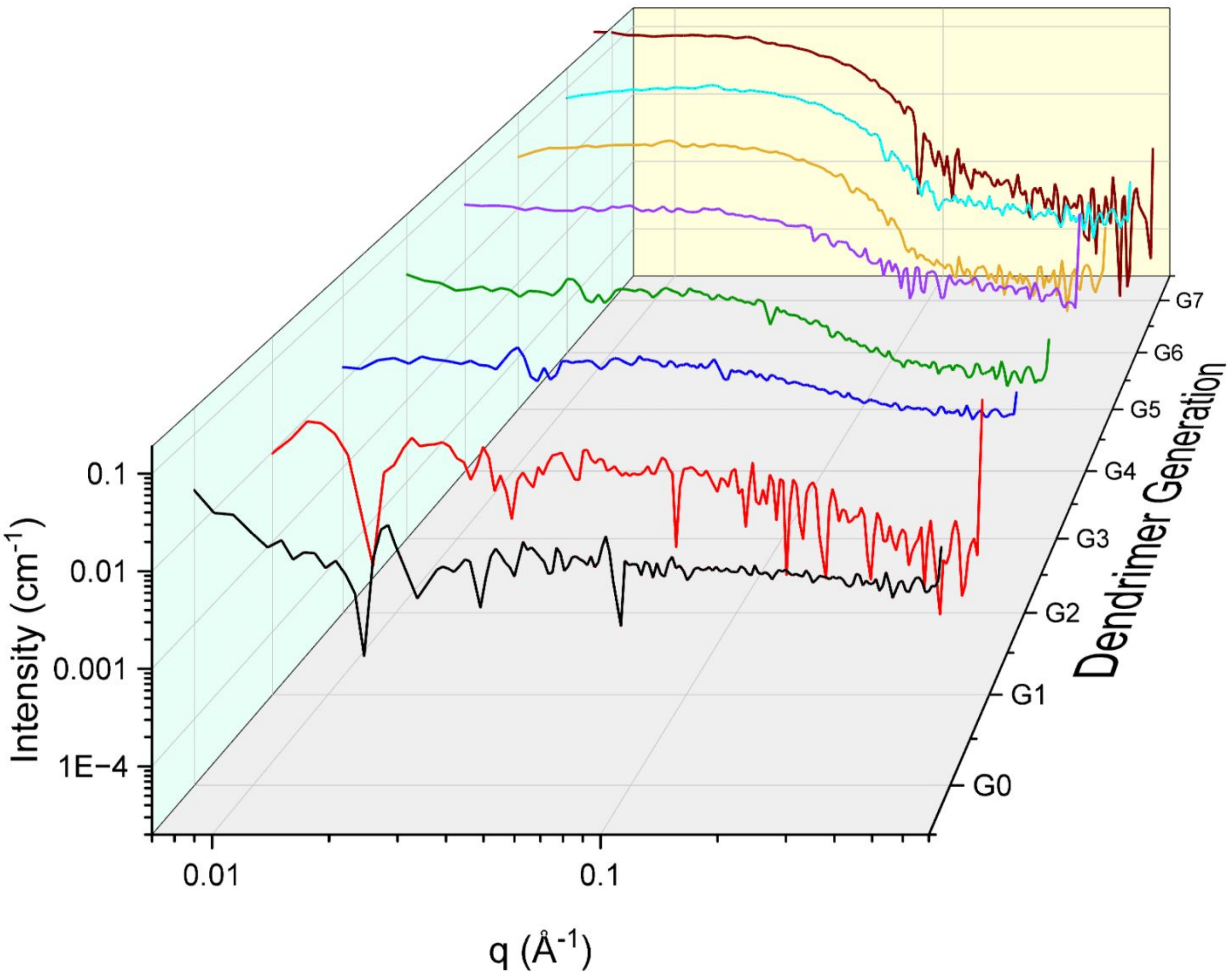

Figure S27 - Waterfall plot of q vs intensity acquired from SANS for PAMAM G0-7
